# Synthesis of the C8’-epimeric thymine pyranosyl amino acid core of amipurimycin

**DOI:** 10.3762/bjoc.12.165

**Published:** 2016-08-05

**Authors:** Pramod R Markad, Navanath Kumbhar, Dilip D Dhavale

**Affiliations:** 1 Garware Research Centre, Department of Chemistry, Savitribai Phule Pune University (Formerly University of Pune), Pune-411007, India

**Keywords:** amipurimycin, 1,3-anhydrosugar, anti-fungal agent, carbohydrate, peptidyl nucleosides

## Abstract

The C8’-epimeric pyranosyl amino acid core **2** of amipurimycin was synthesized from D-glucose derived alcohol **3** in 13 steps and 14% overall yield. Thus, the Sharpless asymmetric epoxidation of allyl alcohol **7** followed by trimethyl borate mediated regio-selective oxirane ring opening with azide, afforded azido diol **10**. The acid-catalyzed 1,2-acetonide ring opening in **10** concomitantly led to the formation of the pyranose ring skeleton to give 2,7-dioxabicyclo[3.2.1]octane **12**. Functional group manipulation in **12** gave **21** that on stereoselective β-glycosylation afforded the pyranosyl thymine nucleoside **2 –** a core of amipurimycin.

## Introduction

**Figure 1:**
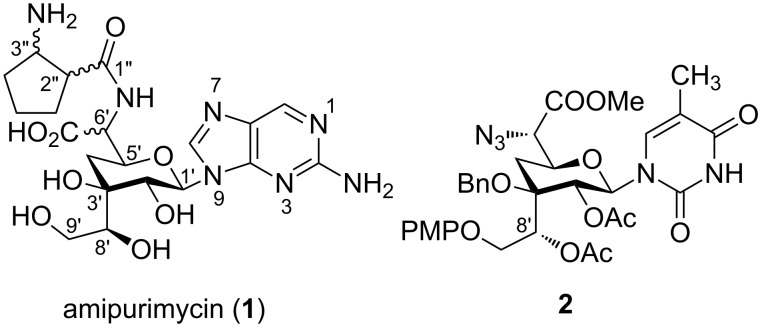
Antifungal antibiotic amipurimycin (**1**).

Peptidyl nucleoside antibiotics are a class of complex molecules that encompass an extensive array of natural products [1]. The notable structural features of peptidyl nucleosides are responsible for their miscellaneous biological activities such as antitumor, antiviral, antibacterial and antifungal [2]. Peptidyl nucleosides in which the sugar part is in the furanose form are common, however, the sugar framework in the pyranose form, with a nucleobase and a peptide linker at either ends, are rare in nature. A few examples of this category are amipurimycin and miharamycin that are known as antifungal agents [2]. Amipurimycin (**1**) isolated from *Streptomyces novoguineensis* sp. nov., displays antifungal activity against *pyricularia oryzae* – a causative agent in rice blast disease [3–4]. Goto and co-workers have proposed the primary structure of amipurimycin (**1**, Figure 1) that involves (a) a unique pyranosyl amino acid ring skeleton with a hydroxy group and a branched 1,2-dihydroxyethyl side chain at C3’, (b) a glycosidic β-linked purine nucleobase, and (c) a five-membered *cis*-pentacin framework coupled to C6’ via the N-terminus of an amino acid [5]. The absolute configurations at the C6’, C2” and C3” of the *cis*-pentacin are still undefined. Thus, the partially unresolved structure, a potent antifungal activity, the unexplored mode of action and the limited synthetic study make amipurimycin (**1**) an attractive target for futher investigation. As of now, a total synthesis of **1** is not known. The reported methodologies are mainly focused on construction of the exigent central pyranosyl amino acid fragment with or without the C3’-branched chain and nucleobase, frequently using carbohydrate substrate [6–15]. In a non-carbohydrate approach, Garner and co-workers have exploited a cycloaddition pathway between a poly-oxygenated diene and Garner’s aldehyde for constructing the carbohydrate core of amipurimycin [9]. Recently, Datta and co-workers reported the first synthesis of a fully-functionalized thymine analogue of amipurimycin, utilizing D-serine as a starting material [6]. In this regard, our group has recently reported the synthesis of the C3’-branched carbohydrate core of amipurimycin starting from D-glucose [15]. In the continuation of this area, we now report the synthesis of the amipurimycin pyranose core comprising of (a) a hydroxy and 1,2-dihydroxyethyl side chain at C3’ having a C8’ epimeric center, (b) an C5’ amino acid pendant and (c) the thymine nucleobase (**2**). Our results in this regard are described herein.

## Result and Discussion

**Scheme 1:**
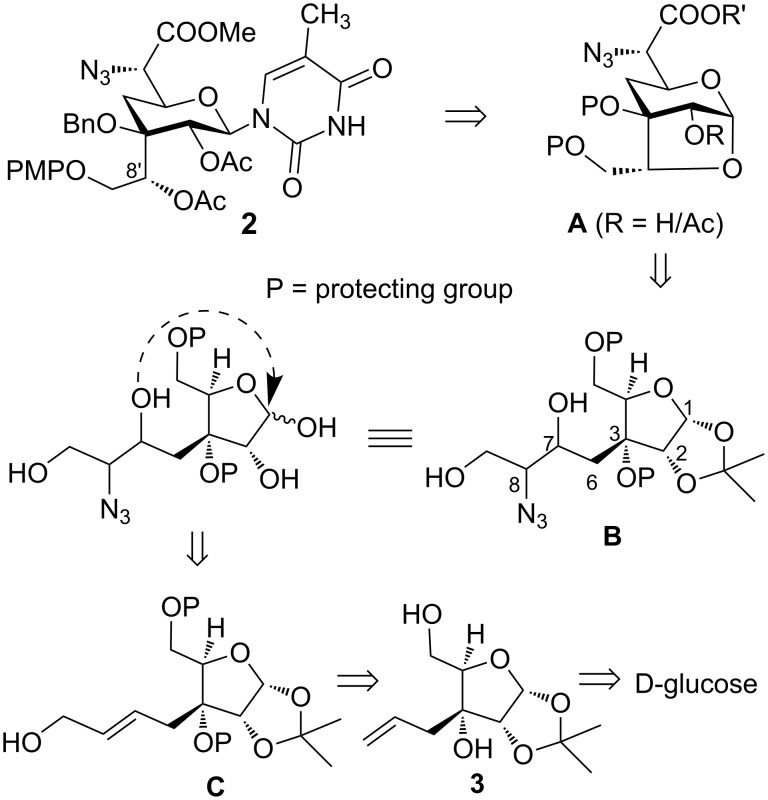
Retrosynthesis of **2**.

As shown in retrosynthetic analysis (Scheme 1), we envisioned that the substituted 2,7-dioxabicyclo[3.2.1]octane **A** could be glycosylated stereoselectively with the requisite nucleobase to give β-nucleoside pyranosyl skeleton **2**. The bridged bicyclic system **A** was visualized from the azido diol **B**. Thus, hydrolysis of the 1,2-acetonide functionality in **B** will lead to in situ generation of oxocarbenium ion at C1 to which concomitant addition of a hydroxy group (present in the side chain at C3) will give the requisite pyranose ring skeleton. Intermediate **B** could be derived from the allyl alcohol **C** by using the Sharpless asymmetric epoxidation followed by regioselective epoxide ring opening with an azide nucleophile. The synthesis of allyl alcohol **C** from D-glucose was reported by us earlier.

**Scheme 2:**
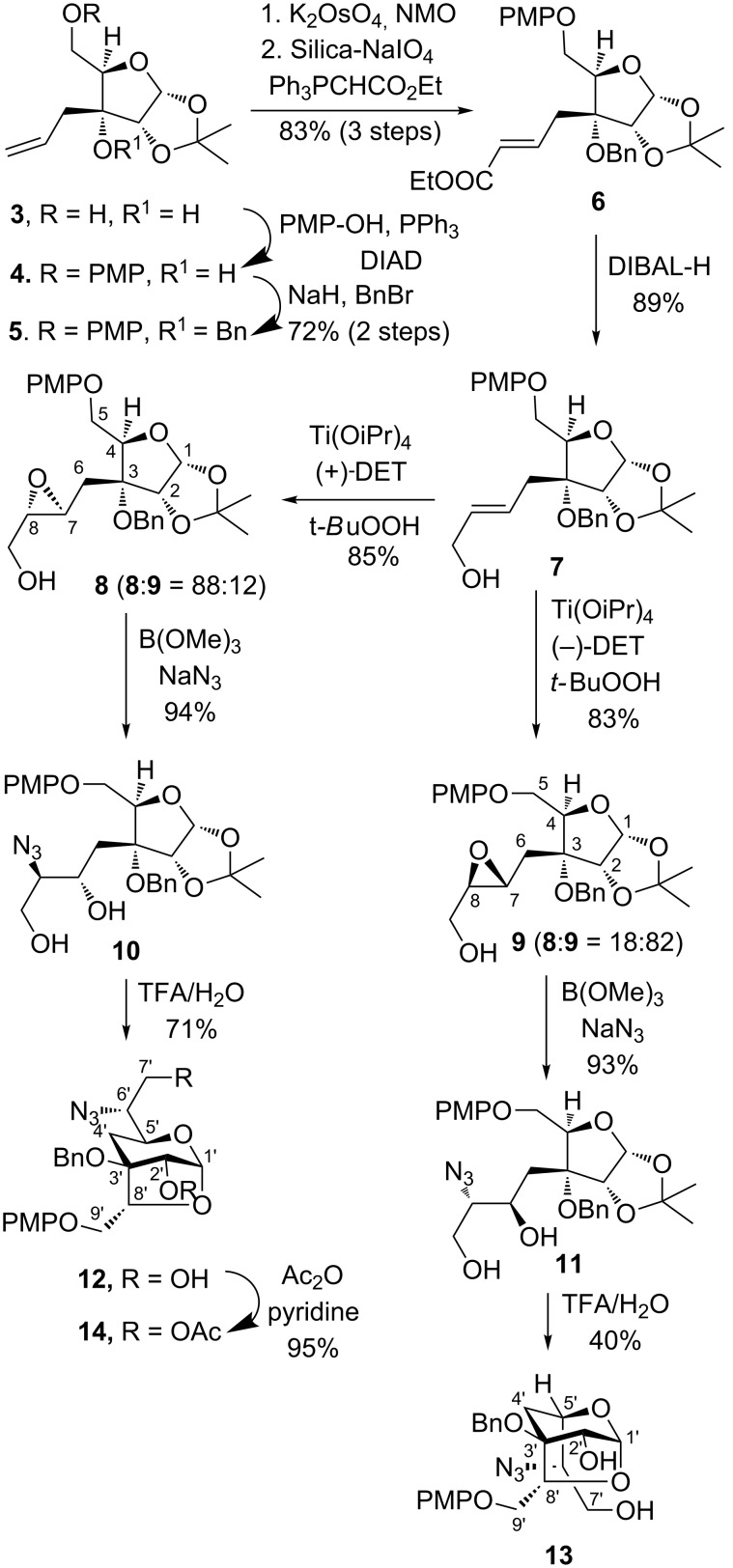
Synthesis of 1,3-anhydrosugar **12** and **13**.

Our synthesis started with the homoallyl alcohol **3** (with defined ‘*R’* absolute configuration at the C3-quaternary center) that is obtained from D-glucose as reported earlier by us in 37% overall yield [15] (Scheme 2). Selective protection of the C5 primary hydroxy group as PMP ether using *p*-methoxyphenol under Mitsunobu reaction conditions afforded **4** that on benzylation of the C3 hydroxy group (NaH and benzyl bromide in DMF) gave compound **5**. Upjohn dihydroxylation of **5** using K_2_OsO_4_·2H_2_O followed by oxidative cleavage of diol with silica-supported NaIO_4_ gave aldehyde that was directly reacted with ethyl 2-(triphenylphosphoranylidene)acetate to give α,β-unsaturated ester **6** (*E*/*Z* = 92:8) in 83% yield over three steps. Reduction of ester **6** (*E*-isomer) with DIBAL-H gave allyl alcohol **7 –** a synthone for the Sharpless asymmetric epoxidation (SAE) [16–17].

Thus, allyl alcohol **7** was subjected for SAE first using (+)-DET that afforded a diastereomeric mixture of epoxy alcohols **8** and **9** in the ratio of 88:12 (based on the ^1^H NMR analysis) in 85% yield. Similarly, use of (–)-DET in SAE afforded epoxide **8** and **9** in the ratio of 18:82 in 83% yield. With the understanding of SAE mnemonic, we assigned the absolute configuration in epoxide **8** as 7*S*,8*S* and in epoxide **9** as 7*R*,8*R*. Subsequently, major isomers of epoxy alcohols **8** and **9** were individually subjected to regioselective epoxide ring opening using trimethyl borate and NaN_3_ in DMF that afforded azido diol **10** and **11** as major isomers, respectively [18–19]. In the next step, individual hydrolysis of the 1,2-acetonide group in **10** and **11** using TFA–H_2_O (3:1) provided the corresponding 1,3-anhydrosugar **12** and **13** in good yield.

**Scheme 3:**
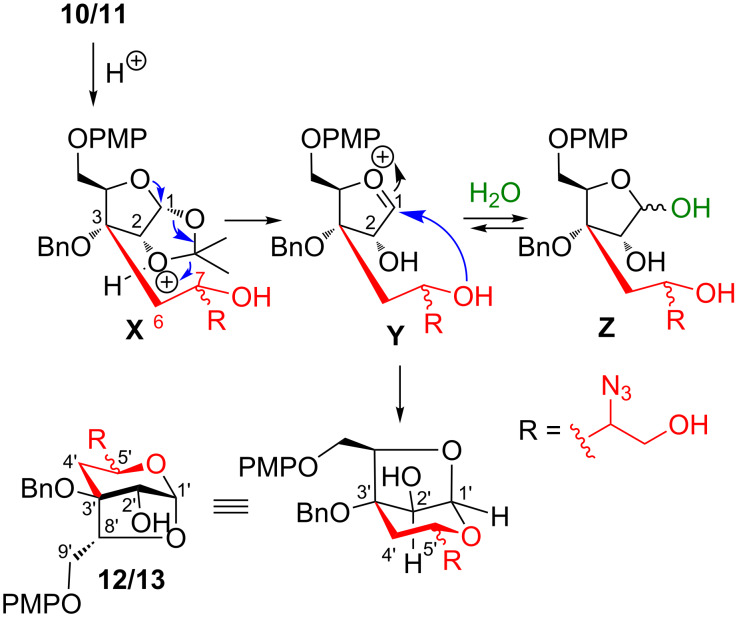
Formation of 2,7-dioxabicyclo[3.2.1]octane **12/13**.

The formation of 1,3-anhydrosugar **12/13** could be explained as follows (Scheme 3). Thus, treatment of **10/11** with TFA–H_2_O resulted in the opening of the 1,2-acetonide functionality and generation of an oxocarbenium ion **Y**. Intermolecular and reversible addition of water would lead to hemiacetal **Z**, however, intramolecular and irreversible attack of the secondary hydroxy group to the oxocarbenium ion **Y** led to a stable six-membered pyranose ring compound thus shifting the equilibrium in favour of bridged bicyclic system **12/13**.

**Figure 2:**
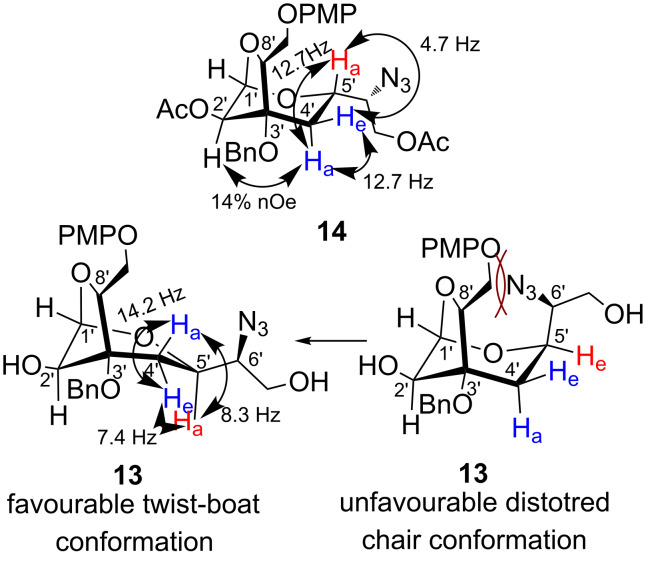
Conformational analysis of **13** and **14**.

In order to validate the configurational assignments at the newly generated stereocenters, the coupling constants of protons at C4’ and C5’ in the pyranose ring of **12**/**13** are noticed to be decisive. In compound **12**, both the C4’ methylene protons were found to be merged and appeared as a multiplet at δ 2.10–2.16 ppm. Therefore, we converted **12** into its acetyl derivative **14** that allowed us to predict the absolute configuration at the newly generated stereocenters at C5’ and C6’ (corresponding to the C7 and C8 of epoxide **8**) [20]. In the ^1^H NMR of **14**, appearance of a triplet at δ 2.30 (*J* = 12.7 Hz) and a doublet of doublet at δ 2.09 (*J* = 12.7 and 4.7 Hz), integrating for one proton each, were assigned to the methylene protons at C4’. Based on the coupling constant and nOe studies (Figure 2), the signal at δ 2.30 was assigned to the axially oriented proton at C4’. As this proton appeared as a triplet with a large coupling constant of 12.7 Hz (vicinal and geminal), the adjacent C5’ proton therefore assigned axial orientation indicating 5’*S* absolute configuration as anticipated from the SAE mnemonic in compound **8**. As the C5’ and C6’ stereocenters in **14** are derived from the regioselective S_N_2 opening of epoxide **8** by NaN_3_, the configuration at the C6’ (carrying azido group) was therefore assigned as 6’*R*.

In the ^1^H NMR spectrum of anhydrosugar **13**, both the C4’ protons appeared as two doublets of doublets at δ 2.47 (*J* = 14.1 and 8.3 Hz) and δ 2.34 (*J* = 14.2 and 7.4 Hz). The vicinal coupling constants of 8.3 and 7.4 Hz between the C4’ and C5’ proton suggested distortion in the conformation of the six-membered ring of the bridged system. Since we have assigned the (5’*S*) configuration at C5’ in **12**, the anhydrosugar **13** expected to have the opposite-5’*R* configuration. Considering this fact, the alkyl pendant (azidoethanol group) in **13** is assumed to be axially oriented. However, this spatial arrangement gives rise to strong steric interactions between the alkyl pendant and C8’ aryloxymethylene group (Figure 2). In order to avoid this strong repulsive interaction, compound **13** adopts relatively more stable twist-boat conformation as shown in Figure 2. The adoption of the twist-boat conformation generates dihedral angles between the H5’ and H4’a/H4’e as ≈150°/25°. This justifies the observed coupling constants in the ^1^H NMR between H5’ and H4’ (*J*_H4’a, H5’a_ = 8.3 Hz and *J*_H4’e, H5’a_ = 7.4 Hz) based on the Karplus equation [21]. This observation also confirms the assigned configurations as 5’*R* and 6’*S* in **13**.

**Figure 3:**
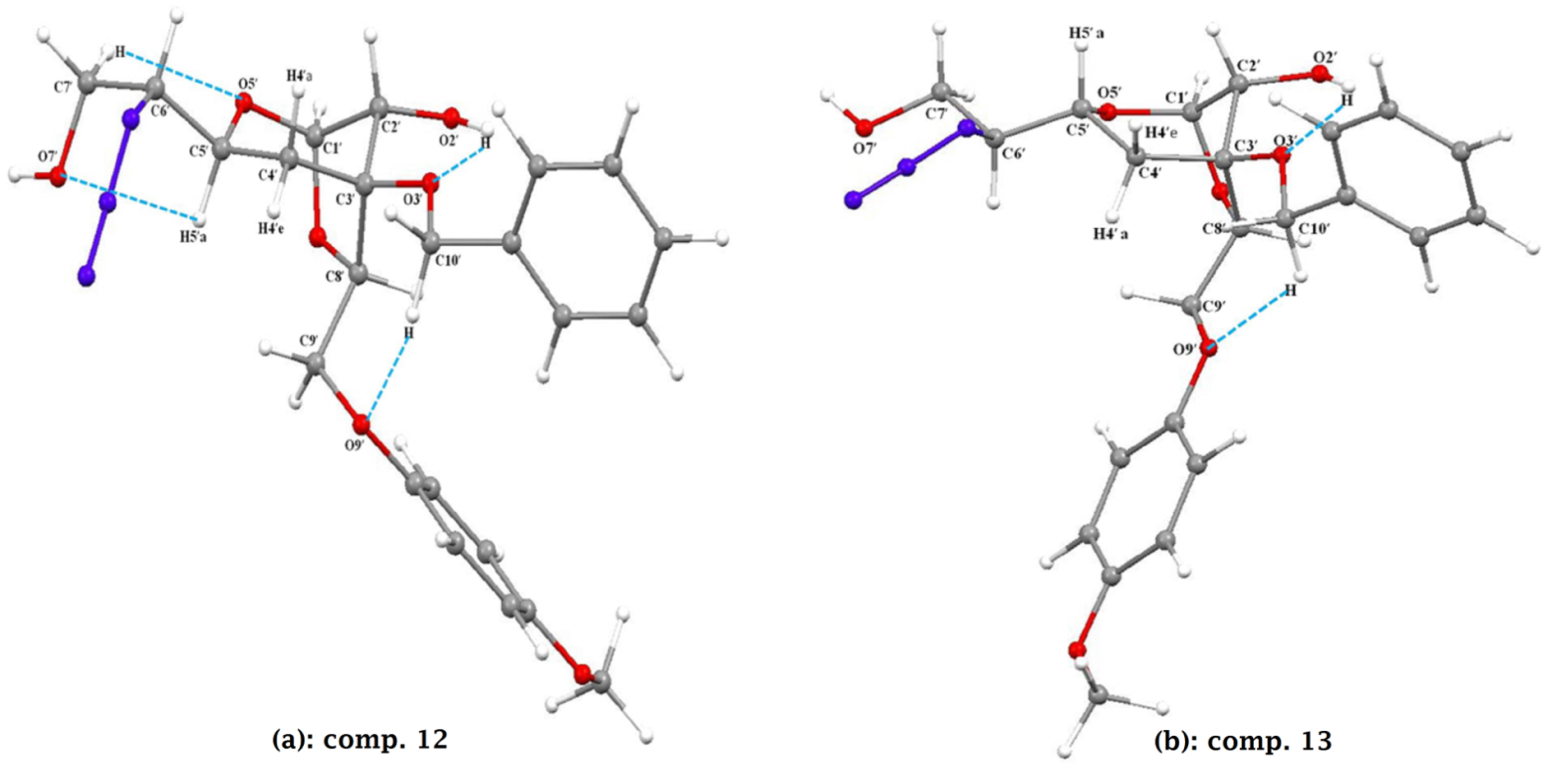
Geometrically optimized conformation of **12** and **13** respectively by DFT study.

The experimental results on conformational preferences of **12** and **13** were corroborated using geometry-optimized density functional theory (DFT). The geometrically optimized preferred conformations of **12** and **13** are depicted in Figure 3, and geometrical parameters for torsion angles and intramolecular hydrogen bonding interactions are given in Table 1. As shown in Figure 3, the preferred conformation of **12** was found to be a stable distorted chair conformation (a). The significant hydrogen bonding interactions between ring oxygen O5’ with H7’ and O7’ with H5’a are accountable for preserving the stable ^4^*C*_1_ chair conformation of compound **12** by maintaining the values 159.77°/42.86° for the θ (H4’a–C4’–C5’–H5’a) and φ (H4’e–C4’–C5’–H5’a) torsion angles (Table 1). Similarly, for compound **13** the preferred conformation was observed to be a twist-boat conformation (Figure 3b). The intramolecular interactions and hydrogen bonding provided additional stability to maintain the twist-boat conformation of **13** (Figure 3) and to adopt the dihedral angles of 143.94°/27.32° between H5’a and H4’a/H4’e, respectively. This supports our earlier assignments made using ^1^H NMR studies.

**Table 1:** Geometrical parameters for hydrogen bonding and torsion angles for **12** and **13**.

Comp.	Atoms involved in H-bonds	Distance in Å	Angle in degree	Torsion angle values	Energy in atomic units (au)

**12**	O5’···H-C7’ O7’···H-C5’ O3’···H-O2’ O9’···H-C10’	2.480 2.550 2.190 2.290	98.74 99.13 112.34 151.77	θ = H4’a–C4’–C5’–H5’ a = 200.23° (−159.77) Φ = H4’e–C4’–C5’–H5’ a = 317.14° (−42.86)	−1583.424 au
**13**	O5’···H-C7’ O3’···H-O2’ O9’···H-C10’	2.426 2.124 2.372	99.68 114.39 122.31	θ = H4’a–C4–C5–H5’ a = 143.94° φ = H4’e–C4–C5–H5’ a = 27.32°	−1583.420 au

**Scheme 4:**
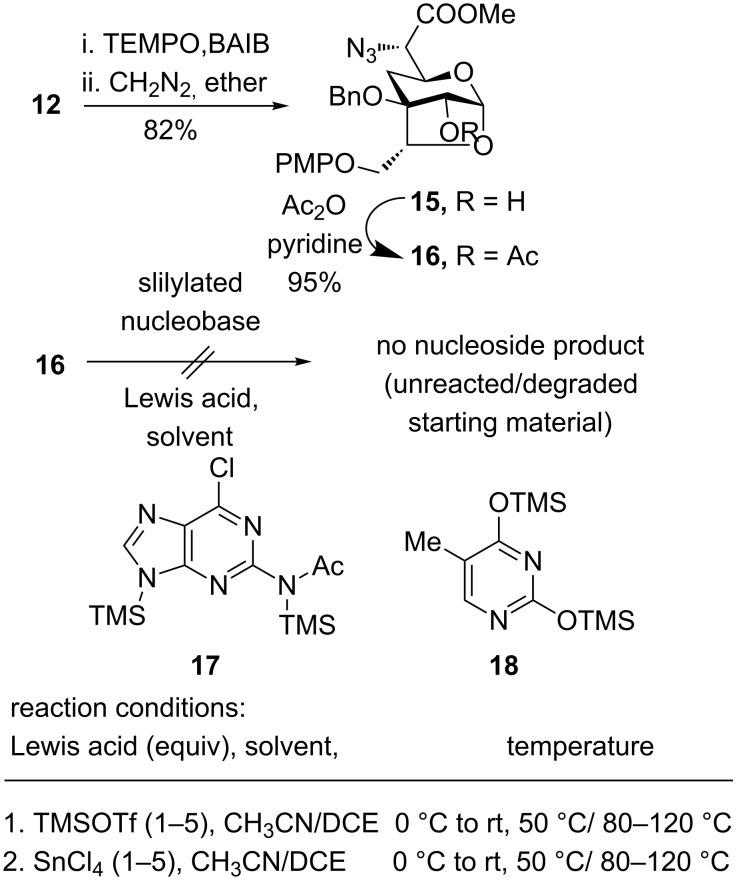
Glycosylation of **16**.

After confirming the absolute configurations of newly generated stereocenters, we continued our synthesis with anhydrosugar **12** as its configurations are matching with that of target compound. Thus, TEMPO-mediated selective oxidation of the primary hydroxy group in **12** to acid functionality followed by esterification using diazomethane afforded azido methyl ester **15**. Acetylation of **15** with acetic anhydride in pyridine gave acetate derivative **16** in 95% yield. Having fully functionalized intermediate **16** in hand, we thought to incorporate the purine nucleobase using Vorbrüggen conditions. Thus, reaction of glycosyl donor **16** with bis(trimethylsilyl)-2-(*N*-acetylamino)-6-chloropurine **17**, under a variety of reaction conditions, of solvents, temperature, Lewis acids as well as the use of the thymine nucleobase **18** (Scheme 4) failed to provide the desired nucleoside.

**Scheme 5:**
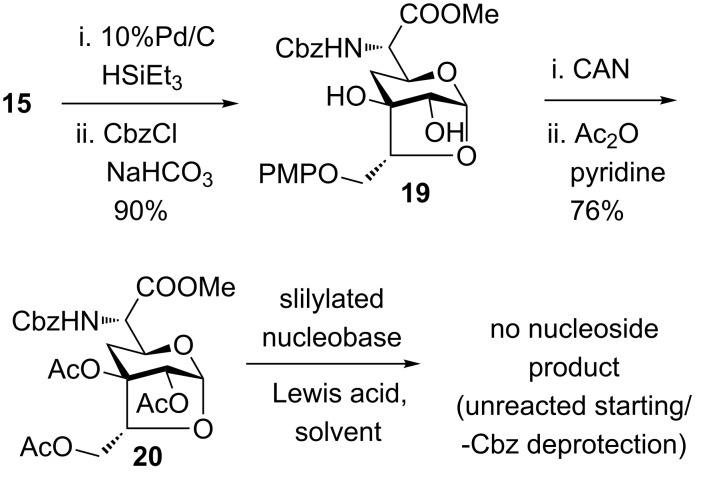
Glycosylation attempt by changing protections.

Knowing the fact that the glycosylation reaction is severely influenced by numerous factors including solvent, Lewis acid, and protecting groups on the nucleobase or sugar; we thought of synthesizing the peracylated anhydrosugar to alter its reactivity towards glycosylation [22]. In this regard, anhydrosugar **15** was subjected to 10% Pd/C and Et_3_SiH (for deprotection of the benzyl functionality and reduction of the azide to an amine) affording a crude product which was directly reacted with CbzCl to afford compound **19** (Scheme 5). Compound **19** on reaction with ceric ammonium nitrate (deprotection of PMP ether) followed by acetyl protection of the resultant triol using Ac_2_O in pyridine gave triacetate derivative **20**. The triacetate **20** was then subjected for glycosylation reaction under similar reaction conditions as above which failed to give the desired product.

**Scheme 6:**
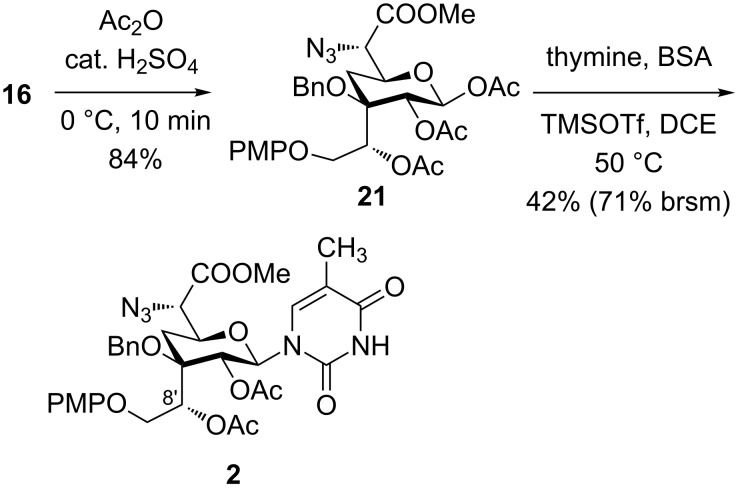
Synthesis of nucleoside **2**.

Alternatively, compound **16** was treated with Ac_2_O with a catalytic amount of H_2_SO_4_ that afforded pyranosyl acetate **21** as an α:β anomeric mixture (1.5:1) in good yield (Scheme 6). Glycosylation of **21** with bis-silylated 2-(*N*-acetylamino)-6-chloropurine under a variety of reaction conditions was found to be unsuccessful. However, glycosylation of **21** with bis(trimethylsilyl)thymine in the presence of TMSOTf in dichloromethane led to the stereoselective formation of β-thymine analogue **2** in 42% (71% based on recovered starting material) yield [23]. In the ^1^H NMR spectrum of **2**, the large coupling constant between H1’ and H2’ (*J* = 9.4 Hz) [5] indicated their relative diaxial orientation confirming the formation of β-glycosylated product **2**. In this reaction, the preferred attack of the thymine nucleobase from the β-face is being assisted by the acetoxy group at C2 due to neighbouring group participation [24]. The orthogonally protected thymine analogue **2** is an important intermediate and could be utilized further for the synthesis of amipurimycin **1** and its analogues for structure–activity relationship (SAR) studies.

## Conclusion

In summary, we have utilized the skeleton of D-glucose-derived homoallyl alcohol **3** as a chiral podium for efficient synthesis of the 8’*R*-glycosyl amino acid core of amipurimycin. With this protocol, we have synthesized the 6’*S* amino acid fragment of amipurimycin. Utilization of a similar protocol with azido diol **11** could be employed for the synthesis of the corresponding 6’*R* fragment. Compound **2** is an important intermediate towards the synthesis of amipurimycin and its analogues for development of SAR and efforts in this regard are in progress.

## Supporting Information

File 1 Experimental procedures.

File 2 Copies of NMR spectra.
