# Supplementary material for: Synthesis of the C8’-epimeric thymine pyranosyl amino acid core of amipurimycin
Source: Beilstein J Org Chem. 2016 Aug 5;12:1765–71. doi: 10.3762/bjoc.12.165 (PMC4979907; doi:10.3762/bjoc.12.165)
Supplement: File 1 — Experimental procedures. [file Beilstein_J_Org_Chem-12-1765-s001.pdf]

Supporting Information
for
Synthesis of the C8’-epimeric thymine pyranosyl amino acid core of amipurimycin

Pramod R. Markad, Navanath Kumbhar and Dilip D. Dhavale*

Address: Garware Research Centre, Department of Chemistry, Savitribai Phule Pune University (Formerly University of Pune), Pune-411007, India.

Email: Dilip D. Dhavale - ddd@chem.unipune.ac.in
*Corresponding author

Experimental procedures

General experimental methods:

Melting points were recorded with Thomas Hoover melting point apparatus and are uncorrected. IR spectra were recorded with FTIR as a thin film or using KBr pellets and are expressed in cm^{-1}. The ^{1}H NMR (300, 400, 500 MHz) and ^{13}C NMR (100, 125 MHz) spectra were recorded using $CDCl_3$ or D_2O as a solvent. Chemical shifts are reported in δ units (ppm) with reference to TMS as an internal standard and *J* values are given in Hz. High resolution mass spectra (HRMS) were obtained in positive ion electrospray (ESI) mode using TOF (time of flight) analyzer. Optical rotations were measured on a digital polarimeter with sodium light (589.3 nm) at 25–30 °C. Thin layer chromatography was performed on pre-coated silica plates. Column chromatography was carried out with silica gel (100–200 mesh). The reactions were carried out in oven-dried glassware under dry N_2. Methanol and THF were purified and dried before use. Distilled *n*-hexane and ethyl acetate were used for column chromatography. After quenching the reaction with water, the work-up involves washing of combined organic layer with water, brine, drying over anhydrous sodium sulphate and evaporation of the solvent at reduced pressure.

1,2-*O*-Isopropylidene-3-*C*-(prop-1'-en-2'-yl)-5-*O*-(4-methoxyphenyl)-α-D-ribofuranose (4)

To a stirred solution of compound **3** (5.00 g, 21.72 mmol) in dry THF (80 mL) under N_2 atmosphere was added triphenylphosphine (8.53 g, 32.58 mmol) and *p*-methoxyphenol (4.04 g, 32.58 mmol) and reaction mixture was cooled to 0 °C. At this temperature DIAD (5.56 mL, 28.23 mmol) was added slowly over a period of 15 min and stirred for 30 min while allowing the reaction to attain room temperature. The reaction mixture was then heated at 80 °C for 2.5 h and cooled to room temperature. Evaporation of the solvent and column chromatography purification (*n*-hexane/ethyl acetate = 9.2/0.8) gave compound **4** (5.70 g, 78.03%) as off white solid: mp = 84–86 °C; R_f 0.6 (*n*-hexane/ethyl acetate = 4/1); $[\alpha]_D^{28.0}$ = 35.51 (*c* 0.41, $CHCl_3$); IR (Neat) 3335, 1640 cm^{-1} ; ^1H NMR (300 MHz, $CDCl_3$) δ 6.89 – 6.76 (m, 4H, Ar), 6.01-5.87 (m, 1H, *H*-7), 5.80 (d, *J* = 3.9 Hz, 1H, *H*-1), 5.28-5.10 (m, 2H, *H*-8), 4.33 (d, *J* = 3.9 Hz, 1H, *H*-2), 4.25-4.14 (m, 2H, *H*-5), 4.05 (dd, *J* = 8.8, 8.0 Hz, 1H, *H*-4), 3.76 (s, 3H, OCH_3), 2.77 (brs, 1H, exchanges with D_2O, O*H*), 2.46 (dd, *J* = 14.5, 6.0 Hz, 1H, *H*-6a), 2.21 (dd, *J* = 14.5, 8.2 Hz, 1H, *H*-6b), 1.59 (s, 3H, CH_3), 1.36 (s, 3H, CH_3). ^{13}C NMR (125 MHz, $CDCl_3$) δ 154.13(Ar), 152.75(Ar), 132.32 (*C*-7), 119.16 (*C*-8), 115.8, 114.6, 112.5 (O-*C*-O), 103.8 (*C*-1), 80.8 (*C*-2), 80.76 (*C*-4), 78.3 (*C*-3), 66.8 (*C*-5), 55.7 (OCH_3), 36.0 (C-6), 26.6 (2 X CH_3). HRMS (ESI-TOF) *m/z*: $[M+Na]^+$ Calcd. for $C_{18}H_{24}NaO_6$ 359.1471; Found 359.1470.

1,2-*O*-Isopropylidene-3-*C*-(prop-1'-en-2'-yl)-3-*O*-benzyl,5-*O*-(4-methoxyphenyl)-α-D-ribofuranose (5)

To a 0 °C cooled suspension of NaH (60% suspension in oil) (0.78 g 19.8 mmol) in dry DMF (5 mL) was added a solution of compound **4** (5.10 g, 15.8 mmol) in dry DMF (10 mL) under N_2 atmosphere and the reaction mixture was stirred for 30 min. To this solution, benzyl bromide

(2.06 mL, 17.38 mmol) was added slowly followed by addition of TBAI (100 mg) and resulting mixture was stirred at rt for 6 h. The reaction mixture was cooled and quenched with drop wise addition of a saturated solution of NH_4Cl in water. Mixture was extracted with ethyl acetate (3 × 50 mL) and worked up. Evaporation of solvent and column chromatography purification (*n*-hexane/ethyl acetate = 19/1) gave compound **5** (6.00 g, 92.2%) as glassy syrup: R_f 0.7 (*n*-hexane/ethyl acetate = 4/1); $[\alpha]_D^{25.0}$ = 22.73 (*c* 2.8, $CHCl_3$); IR (Neat) 1610 cm^{-1}; ^1H NMR (500 MHz, $CDCl_3$) δ 7.5-7.3, 5H, Ar), 6.89 (d, *J* = 9.2 Hz, 2H), 6.84 (d, *J* = 9.2 Hz, 2H), 6.1 – 6.0 (m, 1H, *H*-7), 5.83 (d, *J* = 3.8 Hz, 1H, *H*-1), 5.32 – 5.21 (m, 2H, *H*-8), 4.76 (ABq, *J* = 10.6 Hz, 2H, OCH_2Ph), 4.60 (dd, *J* = 7.7, 2.5 Hz, 1H, *H*-4), 4.51 (d, *J* = 3.8 Hz, 1H, *H*-2), 4.20 – 4.12 (m, 2H, *H*-5), 3.78 (s, 3H, OCH_3), 2.61 (dd, *J* = 15.0, 8.1 Hz, 1H, *H*-6a), 2.55 (dd, *J* = 15.0, 6.0 Hz, 1H, *H*-6b), 1.68 (s, 3H, CH_3), 1.42 (s, 3H, CH_3). ^{13}C NMR (125 MHz, $CDCl_3$) δ 153.95, 152.95, 138.48 (Ar), 132.53 (*C*-7), 128.32, 127.61, 118.76 (*C*-8), 115.63, 114.54, 112.84 (O-*C*-O), 104.29 (*C*-1), 83.66 (*C*-2), 82.23 (*C*-4), 80.08 (*C*-3), 67.43 (OCH_2Ph), 67.04 (*C*-5), 55.69 (OCH_3), 35.69 (*C*-6), 26.93 (CH_3), 26.73 (CH_3). HRMS (ESI-TOF) *m*/*z*: $[M+Na]^+$ Calcd. for $C_{25}H_{30}NaO_6$ 449.1940; Found 449.1945.

1,2-*O*-Isopropylidene-3-*C*-((3'-ethyloxycarbonyl)prop-1'-en-2'-yl)-3-*O*-benzyl-5-*O*-(4-methoxyphenyl)-α-D-ribofuranose (6)

To a solution of **5** (5.50 g, 13.34 mmol) in acetone–water (60 mL, 8:1) was added *N*-methylmorpholine *N*-oxide (3.12 g, 26.69 mmol) and potassium osmate (0.20 g, 5 mol %). The reaction mixture was stirred at room temperature for 24 h. Sodium sulfite (5.00 g) was added to the reaction mixture and stirring continued for 1 h. After removal of acetone under reduced pressure, the residue was extracted with ethyl acetate (50 mL × 4) and concentrated to afford diol as a viscous oil. To the crude diol (5.50 g, 12.55 mmol) in dichloromethane (30 mL) was added

silica supported sodium metaperiodate (10% W/W) (40.00 g, 18.82 mmol) at 0 °C and reaction mixture was stirred for 2 h. After complete consumption of diol (monitored by TLC), ethyl 2-(triphenylphosphoranylidene)acetate (4.35 g, 12.55 mmol) was added to reaction mixture and the reaction mixture was refluxed for 2.5 h. On cooling, reaction mixture was filtered off over celite bed, washed with dichloromethane (15 mL) and filtrate was evaporated under reduced pressure. Purification by column chromatography and first elution with (*n*-hexane/ethyl acetate = 9/1) gave *Z*-isomer *Z*-**6** (0.50 g, 8%) as thick syrup. R_f 0.55 (*n*-hexane/ethyl acetate = 7/3); $[\alpha]_D^{28}$ 37.78 (*c* 0.8, $CHCl_3$); IR (Neat) 1710, 1650 cm^{-1}; ^1H NMR (500 MHz, $CDCl_3$) δ 7.45– 7.30 (m, 5H, Ar), 6.90 – 6.75 (m, 4H, Ar), 6.60 – 6.46 (m, 1H, *H*-7), 5.93 (d, *J* = 11.6 Hz, 1H, *H*-8), 5.85 (d, *J* = 3.8 Hz, 1H, *H*-1), 4.78 (d, *J* = 10.5 Hz, 1H, OCH_a-Ph), 4.67 (d, *J* = 10.6 Hz, 1H, OCH_2Ph), 4.57 (dd, *J* = 7.0, 3.2 Hz, 1H, *H*-4), 4.54 (d, *J* = 3.8 Hz, 1H, *H*-2), 4.21 (q, *J* = 7.1 Hz, 2H, OCH_2-CH_3), 4.15 (dd, *J* = 10.3, 3.2 Hz, 1H, *H*-5a), 4.06 (dd, *J* = 10.3, 7.0 Hz, 1H, *H*-5b), 3.75 (s, 3H, OCH_3), 3.42 (dd, *J* = 18.0, 7.8 Hz, 1H, *H*-6a), 2.99 (dd, *J* = 18.0, 5.3 Hz, 1H, *H*-6b), 1.66 (s, 3H, CH_3), 1.39 (s, 3H, CH_3), 1.32 (t, *J* = 7.1 Hz, 3H, CH_2-CH_3). ^{13}C NMR (125 MHz, $CDCl_3$) δ 166.26 (*C*OO), 153.99, 152.74, 143.25 (*C*-7), 138.24, 128.31, 127.62, 127.55, 121.74 (*C*-8), 115.60, 114.53, 112.91(O-*C*-O), 104.13 (*C*-1), 83.87 (*C*-2), 82.60 (*C*-3), 79.32 (*C*-4), 67.02 (OCH_2-Ph), 66.75 (*C*-5), 60.20 (OCH_2CH_3), 55.69 (OCH_3), 29.96 (*C*-6), 26.86 (CH_3), 26.67 (CH_3), 14.26 (CH_2CH_3). HRMS (ESI-TOF) *m*/*z*: $[M+Na]^+$ Calcd for $C_{28}H_{34}NaO_8$ 521.2151; Found 521.2145.

Further elution with (*n*-hexane/ethyl acetate = 22/3) gave *E*-**6** (5.36 g, 83.1% over three steps) as a white solid: mp = 81–82° °C; R_f 0.5 (*n*-hexane/ethyl acetate = 7/3); $[\alpha]_D^{28}$ 43.20 (*c* 0.5, $CHCl_3$); IR (Neat) 1704, 1654 cm^{-1}; ^1H NMR (500 MHz, $CDCl_3$) δ 7.45-7.2, m, 5H, Ar), 7.11 (dt, *J* = 15.5, 7.3 Hz, 1H, *H*-7), 6.82 (d, *J* = 9.3 Hz, 2H, Ar), 6.79 (d, *J* = 9.2 Hz, 2H, Ar), 5.97 (d, *J* =

15.7 Hz, 1H, *H*-8), 5.79 (d, *J* = 3.7 Hz, 1H, *H*-1), 4.77 (d, *J* = 10.5 Hz, 1H, CH_2Ph), 4.69 (d, *J* = 10.5 Hz, 1H, CH_2Ph), 4.56 (dd, *J* = 7.2, 2.9 Hz, 1H, *H*-4), 4.47 (d, *J* = 3.7 Hz, 1H, *H*-2), 4.19 (q, *J* = 7.1 Hz, 2H, OCH_2CH$_3$), 4.13 (dd, *J* = 10.3, 2.9 Hz, 1H, *H*-5a), 4.06 (dd, *J* = 10.3, 7.2 Hz, 1H, *H*-5b), 3.75 (s, 3H, OCH_3), 2.70 (dd, *J* = 15.3, 8.1 Hz, 1H, *H*-6a), 2.60 dd, *J* = 15.3, 6.5 Hz, 1H, *H*-6b), 1.64 (s, 3H, CH_3), 1.38 (s, 3H, CH_3), 1.28 (t, *J* = 7.1 Hz, 3H, CH$_2$CH_3). ^{13}C NMR (125 MHz, CDCl$_3$) δ 165.96 (*C*OO), 154.16, 152.80, 142.50 (*C*-7), 138.20, 128.48, 127.86, 127.77, 124.92 (*C*-8), 115.72, 114.67, 113.22 (O-*C*-O), 104.10 (*C*-1), 83.91 (*C*-2), 82.32 (*C*-3), 79.48 (*C*-4), 67.52 (O*C*H$_2$-Ph), 66.92 (*C*-5), 60.65 (O*C*H$_2$CH$_3$), 55.81 (O*C*H$_3$), 34.16 (*C*-6), 26.95 (*C*H$_3$), 26.80 (*C*H$_3$), 14.40 (CH$_2$*C*H$_3$). HRMS (ESI-TOF) *m*/*z*: [M+Na]$^+$ Calcd. for $C_{28}H_{34}NaO_8$ 521.2151; Found 521.2145.

1,2-*O*-Isopropylidene-3-(*E*-but-2'-en-4-ol)-3-*O*-benzyl-5-*O*-(4-methoxyphenyl)-α-D-ribofuranose (7)

To a cooled solution of **6** (2.00 g, 4.01 mmol) in dry dichloromethane (30 mL) at −30 °C was added DIBAL-H (4.41 mL, 8.8 mmol, 2M solution in toluene) and reaction mixture was stirred at −30 °C for 6 h. The reaction was quenched with a saturated solution of Rochelle's salt and the resulting complex was allowed to attain 30 °C and wasstirred for 4 h to get two distinct phases. The organic phase was then separated and worked up. Evaporation of dichloromethane under reduced pressure and purification by column chromatography (*n*-hexane/ethyl acetate = 7/3) gave **7** (1.63 g, 89.21%) as viscous oil: R_f 0.5 (*n*-hexane/ethyl acetate = 1/1); $[\alpha]_D^{28}$ 22.53 (*c* 2.0, CHCl$_3$); IR (Neat) 3600–3200 (broad) 1665 cm^{-1}; ^{1}H NMR (300 MHz, CDCl$_3$) δ 7.5-7.25 (m, 5H, Ar), 6.85 – 6.77 (m, 4H, Ar), 5.87 – 5.74 (m, 2H, *H*-1, *H*-8), 4.76- 4.65 (m, 3H, *H*-7, OCH_2Ph), 4.55 (dd, *J* = 7.0, 3.5 Hz, 1H, *H*-4), 4.46 (d, *J* = 3.8 Hz, 1H, *H*-2), 4.15 – 4.00 (m, 3H, 2 X *H*-5, *H*-9a), 3.74 (s, 4H, *H*-9b, OCH_3), 2.60 – 2.46 (m, 2H, *H*-6), 1.87 – 1.81 (m, 1H,

exchanges with D_2O, OH), 1.62 (s, 3H, CH_3), 1.36 (s, 3H, CH_3); ^{13}C NMR (125 MHz, $CDCl_3$) δ 153.98, 152.79, 138.41, 133.08 (*C*-7), 128.30, 127.60, 127.50, 125.90 (*C*-8), 115.57, 114.58, 112.92 (O-*C*-O), 104.12 (*C*-1), 83.72 (*C*-3), 82.38 (*C*-2), 79.79 (*C*-4), 67.10 (*C*-5), 67.00 (*C*-9), 63.11 (O*C*H_2Ph), 55.70 (O*C*H_3), 34.00 (*C*-6), 26.88 (*C*H_3), 26.69 (*C*H_3). HRMS (ESI-TOF) *m*/*z*: $[M+Na]^+$ Calcd for $C_{26}H_{32}NaO_7$ 479.2045; Found 479.2040.

1,2-*O*-Isopropylidene-3-*C*-(2'*S*-3'*S*-hydroxymethyloxiran-2-ylmethyl)-3-*O*-benzyl-5-*O*-(4-methoxyphenyl)-α-D-ribofuranose (8)

To a solution of titanium isopropoxide (0.97 mL, 3.29 mmol) in dry dichloromethane (5 mL) and powdered 4 Å molecular sieves (2.00 g), was added (+)-diethyl tartrate (0.62 mL, 3.61 mmol) at −20 °C. To this solution, compound **7** (1.5 g, 3.29 mmol) in dichloromethane (10 mL) was added slowly (15 min) and stirred for 30 min. Then, *t*-BuOOH (1.31 mL, 6.57 mmol, 5–6 M in decane) was added. The reaction mixture was stirred for 15 min. and kept in a refrigerator at (−5 °C) for 12 h. After completion of the reaction, a saturated solution of sodium sulfate (2 mL) and diethyl ether (10 mL) was added and stirred for 15 min, filtered over a celite bed and washed with diethyl ether (10 mL). Evaporation of the solvent and purification of crude compound by column chromatography (*n*-hexane/ethyl acetate = 7/3) gave major isomer **8** (1.33 g, 85.66%) as viscous oil: R_f 0.45 (*n*-hexane/ethyl acetate = 1/1); $[\alpha]_D^{28}$ 7.25 (*c* 1.25, $CHCl_3$); ^{1}H NMR (400 MHz, $CDCl_3$) δ 7.5−7.25 (m, 5H, Ar), 6.96 – 6.75 (m, 4H, Ar), 5.96 (d, *J* = 3.8 Hz, 1H, *H*-1), 4.77 (d, *J* = 10.8 Hz, 1H, CH_2Ph), 4.65–4.61 (m, 2H, *H*-2 and CH_2Ph), 4.59 (dd, *J* = 6.6, 3.0 Hz, 1H, *H*-4), 4.23 (m, 2H, *H*-5), 3.86 (dd, *J* = 12.6, 2.8 Hz, 1H, *H*-9a), 3.77 (s, 3H, OCH_3) 3.61 (dd, *J* = 12.6, 4.6 Hz, 1H, *H*-9b), 3.32 (dt, *J* = 8.0, 2.5 Hz, 1H, *H*-7), 2.95 (dt, *J* = 4.8, 2.6 Hz, 1H, *H*-8), 2.22 (dd, *J* = 15.0, 2.7 Hz, 1H, *H*-6a), 1.92 (t, *J* = 6.0 Hz, 1H, exchanges with D_2O, O*H*), 1.84 (dd, *J*

= 15.0, 8.2 Hz, 1H, *H*-6b), 1.67 (s, 3H, C*H*$_3$), 1.41 (s, 3H, C*H*$_3$). ^{13}C NMR (100 MHz, CDCl$_3$) δ 154.03, 152.80, 138.31, 128.32, 127.88, 127.65, 127.52, 115.69, 115.63, 114.61, 112.90 (O-*C*-O), 104.37 (*C*-1), 83.88 (*C*-2), 82.26 (*C*-3), 79.62 (*C*-4), 67.16 (O-*C*H$_2$Ph), 66.69 (*C*-5), 61.57 (*C*-9), 58.76 (*C*-7), 55.70 (O*C*H$_3$), 51.36 (*C*-8), 34.20 (*C*-6), 26.79 (*C*H$_3$), 26.60 (*C*H$_3$). HRMS (ESI-TOF) *m*/*z*: [M+Na]$^+$ Calcd for $C_{26}H_{32}NaO_8$ 495.1995; Found 495.1989.

1,2-*O*-Isopropylidene-3-*C*-(2'*R*-3'*R*-hydroxymethyloxiran-2-ylmethyl)-3-*O*-benzyl,5-*O*-(4-methoxyphenyl)-α-D-ribofuranose (9)

Reaction of **7** (0.2 g, 0.44 mmol) with Ti(O-iPr)$_4$ (0.13 mL, 0.44 mmol), D-(−) diethyl tartarate (0.082 mL, 0.48 mmol), *t*-BuOOH (0.175 mL, 0.88 mmol, 5–6 M in decane,), as described earlier for **8,** gave major isomer **9** as thick liquid (0.172 g, 83.3%): R_f 0.45 (*n*-hexane/ethyl acetate = 1/1); $[\alpha]_D^{25}$ 16.26 (*c* 0.5, CHCl$_3$); ^{1}H NMR (300 MHz, CDCl$_3$) δ 7.45 – 7.26 (m, 5H, Ar), 6.87 – 6.75 (m, 4H, Ar), 5.81 (d, *J* = 3.6 Hz, 1H, *H*-1), 4.90 – 4.53 (m, 4H, OC*H*$_2$Ph, *H*-2, *H*-4), 4.22 – 4.03 (m, 2H, *H*-5), 3.80 (dd, *J* = 12.7, 2.6 Hz, 1H, *H*-9a'), 3.75 (s, 3H, OC*H*$_3$), 3.56 (dd, *J* = 12.7, 4.2 Hz, 1H, *H*-9b'), 3.35 – 3.22 (m, 1H, *H*-8'), 2.94 (m, 1H, *H*-7'), 2.20 (dd, *J* = 15.1, 3.4 Hz, 1H, *H*-6a'), 1.72 (dd, *J* = 15.2, 6.9 Hz, 1H, *H*-6b'), 1.65 (s, 3H, *C*H$_3$), 1.39 (s, 3H, *C*H$_3$). ^{13}C NMR (125 MHz, CDCl$_3$) δ 154.00, 152.77, 138.27, 128.34, 127.67, 127.54, 115.66, 115.60, 114.62, 114.59 (Ar), 112.91 (O-*C*-O), 104.36 (*C*-1), 83.87 (*C*-2), 82.22 (*C*-3), 79.63 (*C*-4), 67.16 (O*C*H$_2$Ph), 66.66 (*C*-5), 61.47 (*C*-9'), 58.81(*C*-7'), 55.70 (O*C*H$_3$), 51.38 (*C*-8'), 34.15 (*C*-6'), 26.78 (*C*H$_3$), 26.58 (*C*H$_3$). HRMS (ESI-TOF) *m*/*z*: [M+Na]$^+$ Calcd for $C_{26}H_{32}NaO_8$ 495.1995; Found 495.1989.

1,2-*O*-Isopropylidene-3-*C*-(3'*R*-azido-2'*S*-hydroxybutanol)-3-*O*-benzyl-5-*O*-(4-methoxyphenyl)-α-D-ribofuranose (10)

To a solution of compound **8** (1.00 g, 2.12 mmol) in dry DMF (10 mL) was added NaN_3 (0.34 g, 5.29 mmol) followed by addition of $B(OMe)_3$ (0.50 mL, 3.17 mmol). The reaction mixture was stirred at 50 °C for 3 h. After completion, the reaction mixture was allowed to attain rt and quenched with a saturated solution of $NaHCO_3$ (10 mL) and stirred for 10 min. The aqueous phase was extracted with ethyl acetate (3 × 15 mL), worked up and the ethyl acetate was evaporated under reduced pressure. Purification by column chromatography (*n*-hexane/ethyl acetate = 7/3) gave **10** (1.04 g, 95.32%) as viscous oil: R_f 0.55 (*n*-hexane/ethyl acetate = 1/1); $[\alpha]_D^{28}$ 12.71 (*c* 2.0, $CHCl_3$); IR (Neat) 3600–3400 (broad), 2120 cm^{-1}; ^1H NMR (500 MHz, $CDCl_3$) δ 7.5 – 7.29 (m, 5H, Ar), 6.95 – 6.81 (m, 4H, Ar), 5.81 (d, *J* = 3.7 Hz, 1H, *H*-1), 5.12 (d, *J* = 10.3 Hz, 1H, OCH_2-Ph), 4.92 (t, *J* = 5.4 Hz, 1H, *H*-4), 4.87 (d, *J* = 10.3 Hz, 1H, OCH_2-Ph), 4.71 (d, *J* = 3.7 Hz, 1H, *H*-2), 4.15 (d, *J* = 5.4 Hz, 2H, *H*-5), 4.13-4.10 (m, 1H, *H*-7), 3.93 – 3.88 (m, 2H, *H*-9), 3.79 (s, 3H, OCH_3), 3.35 (dt, *J* = 7.6, 4.9 Hz, 1H, *H*-8), 2.44 (t, *J* = 5.1 Hz, 1H, exchanges with D_2O, O*H*), 1.94 (dd, *J* = 14.5, 9.7 Hz, 1H, *H*-6a), 1.85 (d, *J* = 14.5 Hz, 1H, *H*-6b), 1.70 (s, 3H, CH_3), 1.66 (brs, 1H, exchanges with D_2O, O*H*), 1.42 (s, 3H, CH_3). ^{13}C NMR (125 MHz, $CDCl_3$) δ 154.26, 152.59, 137.95, 128.64, 128.07, 127.92, 115.82, 115.74,114.68 (Ar), 113.16 (O-*C*-O), 103.66 (*C*-1), 103.47, 84.65 (*C*-2), 82.58 (*C*-4), 75.41(*C*-3), 69.03 (*C*-7), 68.63 (OCH_2Ph), 66.70 (*C*-5), 66.50 (*C*-9), 62.68 (*C*-8), 55.73 (OCH_3), 35.52 (*C*-6), 26.78 (CH_3), 26.33 (CH_3). HRMS (ESI-TOF) *m*/*z*: $[M+Na]^+$ Calcd for $C_{26}H_{33}N_3NaO_8$ 538.2160; Found 538.2163.

1,2-*O*-Isopropylidene-3-*C*-(3'*S*-azido-2'*R*-hydroxybutanol)-3-*O*-benzyl-5-*O*-(4-methoxyphenyl)-α-D-ribofuranose (**11**)

Reaction of **9** (0.15 g, 0.32 mmol) with NaN_3 (0.041 g, 0.63 mmol) and $B(OMe)_3$ (0.053 mL, 0.48 mmol) in DMF, as described for **10**, gave **11** as thick oil (0.152 g, 93.2%); R_f 0.55 (*n*-hexane/ethyl acetate = 1/1); $[\alpha]_D^{25}$ 22.28 (*c* 2.09, $CHCl_3$); IR (Neat) 3600–3400 (broad), 2121 cm^{-1}; ^1H NMR (500 MHz, $CDCl_3$) δ 7.45 – 7.30 (m, 5H, Ar), 6.89 – 6.82 (m, 4H, Ar), 5.82 (d, *J* = 3.65 Hz, 1H, *H*-1), 4.97 (d, *J* = 10.5 Hz, 1H, OCH_2-Ph), 4.86 (d, *J* = 10.5 Hz, 1H, OCH_2-Ph), 4.79 (dd, *J* = 6.2, 4.6 Hz, 1H, *H*-4), 4.74 (d, *J* = 3.65 Hz, 1H, *H*-2), 4.21 – 4.11 (m, 2H, *H*-5), 4.05–4.0 (m, 1H, *H*-7'), 3.82 (m, 1H, *H*-9a'), 3.80 – 3.73 (m, 4H, H-9b', OCH_3), 3.39 (dd, *J* = 11.5, 5.1 Hz, 1H, *H*-8'), 2.12 (dd, *J* = 15, 1.2 Hz, 1H, *H*-6a'), 1.78 (dd, *J* = 15.0, 9.7 Hz, 1H, *H*-6b'), 1.66 (s, 3H, CH_3), 1.40 (s, 3H, CH_3). ^{13}C NMR (125 MHz, $CDCl_3$) δ 154.25, 152.38, 138.15, 128.39, 127.80, 115.73, 114.64 (Ar), 113.25 (O-*C*-O), 103.45 (*C*-1), 84.02 (*C*-2), 82.58 (*C*-4), 75.35 (*C*-3), 69.06 (OCH_2-Ph), 68.65 (*C*-5), 66.87 (*C*-7'), 66.62 (*C*-9'), 62.44 (*C*-8'), 55.71 (OCH_3), 35.25 (*C*-6'), 26.79 (CH_3), 26.55 (CH_3). HRMS (ESI-TOF) *m*/*z*: $[M+Na]^+$ Calcd for $C_{26}H_{33}N_3NaO_8$ 538.2160; Found 538.2163.

(1*R*,3*S*,5*S*,6*R*,8*R*)-3-((*R*)-1-azido-2-hydroxyethyl)-5-(benzyloxy)-6-((4-methoxyphenoxy)-methyl)-2,7-dioxabicyclo[3.2.1]octan-8-ol (**12**)

A solution of **10** (0.50 g, 0.97 mmol) in TFA–water (5.0 mL, 3:1) was stirred for 1 h at 0 °C then for 2 h at 25 °C. TFA was co-evaporated with toluene at reduced pressure. Purification by column chromatography with (*n*-hexane/ ethylacetate = 4/1) furnished **12** (0.315 g, 71.03%) as viscous liquid: R_f 0.45 (*n*-hexane/ethyl acetate = 3/2); $[\alpha]_D^{28}$ 54.25 (*c* 0.93, $CHCl_3$); IR (Neat) 3600–3200 (broad), 2120 cm^{-1}; ^1H NMR (500 MHz, $CDCl_3$) δ 7.40 – 7.32 (m, 5H, Ar), 6.93 –

6.84 (m, 4H, Ar), 5.34 (s, 1H, *H*-1'), 4.72 (d, *J* = 11.0 Hz, 1H, C*H*$_2$-Ph), 4.63 (d, *J* = 5.0 Hz, 1H, *H*-8'), 4.59 (d, *J* = 11.0 Hz, 1H,C*H*$_2$-Ph), 4.35 – 4.31 (m, 1H, *H*-5'), 4.30 (d, *J* = 5.0 Hz, 2H, *H*-9'), 3.91 (s, 1H, *H*-2'), 3.78-3.72 (m, 4H, OCH$_3$, *H*-6'), 3.71 – 3.65 (m, 2H, *H*-7'), 2.16 – 2.10 (m, 2H, *H*-4'), 1.65 (brs, 2H, exchanges with D$_2$O, 2 x O*H*). ^{13}C NMR (125 MHz, CDCl$_3$) δ 154.19 (Ar), 152.55 (Ar), 137.45 (Ar), 128.75 (Ar), 128.26 (Ar), 127.55 (Ar), 115.59 (Ar), 114.69 (Ar), 102.32 (*C*-1'), 80.97 (*C*-2'), 79.35 (*C*-8'), 76.39 (*C*-3'), 69.57 (*C*H$_2$-Ph), 67.04 (*C*-5'), 66.42 (*C*-9'), 66.21 (*C*-7'), 62.50 (*C*-6'), 55.74 (O*C*H$_3$), 31.64 (*C*-4'). HRMS (ESI-TOF) *m*/*z*: [M+Na]$^+$ Calcd for $C_{23}H_{27}N_3NaO_7$ 480.1747; Found 480.1736.

(1*R*,3*S*,5*S*,6*R*,8*R*)-3-((*R*)-1-Azido-2-hydroxyethyl)-5-(benzyloxy)-6-((4-methoxyphenoxy) methyl)-2,7-dioxabicyclo[3.2.1]octan-8-ol (13)

Reaction of **11** (0.05 g, 0.097 mmol) with TFA-H$_2$O, as described for **12**, gave **13** as thick liquid (0.017 g, 40.3%): *R*$_f$ 0.35 (*n*-hexane/ethyl acetate = 3/2); $[\alpha]_D^{28}$ 55.21 (*c* 0.41, CHCl$_3$); IR (Neat) 3600–3200 (broad), 2120 cm^{-1}; ^{1}H NMR (500 MHz, CDCl$_3$) δ 7.45 – 7.28 (m, 5H, Ar), 6.92 – 6.82 (m, 4H, Ar), 5.28 (s, 1H, *H*-1'), 4.75 (d, *J* = 11.0 Hz, 1H, OC*H*$_2$-Ph), 4.62 (d, *J* = 11.0 Hz, 1H, OC*H*$_2$-Ph), 4.54 (t, *J* = 5.5 Hz, 1H, *H*-8'), 4.30-4.23 (m, 3H, *H*-7a', 2 x *H*-9') 4.07 (m, 1H, *H*-5'), 3.85 (dd, *J* = 11.6, 4.1 Hz, 1H, *H*-7b'), 3.78 – 3.73 (m, 4H, *H*-2, OC*H*$_3$), 3.64 (dd, *J* = 10.8, 6.0 Hz, 1H, *H*-6'), 2.47 (dd, *J* = 14.2, 8.3 Hz, 1H, *H*-4a'), 2.34 (dd, *J* = 14.2, 7.4 Hz, 1H, *H*-4b'), 1.61 (brs, 2H, exchanges with D$_2$O, 2 x O*H*). ^{13}C NMR (125 MHz, CDCl$_3$) δ 154.19, 152.54, 137.48, 128.69, 128.16, 127.54, 115.58, 114.73 (Ar), 101.81 (*C*-1'), 80.94 (*C*-2'), 78.15 (*C*-8'), 72.89 (*C*-3'), 68.05 (*C*-5'), 67.83 (O*C*H$_2$Ph), 67.22 (*C*-9'), 66.39 (*C*-7'), 62.18 (*C*-6'), 55.74 (O*C*H$_3$), 30.60 (*C*-4'). HRMS (ESI-TOF) *m*/*z*: [M+Na]$^+$ Calcd for $C_{23}H_{27}N_3NaO_7$ 480.1747; Found 480.1735.

(1*R*,3*S*,5*R*,6*R*,8*R*)-3-((*R*)-2-Acetoxy-1-azidoethyl)-5-(benzyloxy)-6-((4-methoxyphenoxy) methyl)-2,7-dioxabicyclo[3.2.1]octan-8-yl acetate (14)

To a cooled solution of compound **12** (0.10 g, 0.22 mmol) in pyridine (1 mL) was added Ac_2O (0.62 mL, 0.66 mmol) at 0 °C. The reaction mixture was stirred for 3 h while attaining 25 °C. Dichloromethane (10 mL) was added to the reaction mixture and washed with 10% HCl solution (2 × 05 mL). Work up and evaporation of solvent at reduced pressure and purification by column chromatography using *n*-hexane/ethyl acetate = 9/1 furnished **14** (0.113 g, 95.46%) as white solid: R_f 0.45 (*n*-hexane/ethyl acetate = 3/2); mp = 76–78 °C; $[\alpha]_D^{25}$ 39.55 (*c* 1.08, $CHCl_3$); IR (Neat) 2118 and 1738 cm^{-1}; ^1H NMR (500 MHz, $CDCl_3$) δ 7.40 – 7.29 (m, 5H, Ar), 6.92 (d, *J* = 9.2 Hz, 2H, Ar), 6.85 (d, *J* = 9.2 Hz, 2H, Ar), 5.40 (s, 1H, *H*-1'), 5.09 (s, 1H, *H*-2'), 4.64 (d, *J* = 10.8 Hz, 1H, -OCH_2-Ph), 4.63 (m, 1H, *H*-8'), 4.53 (d, *J* = 10.8 Hz, 1H, -OCH_2-Ph), 4.32 (dd, *J* = 11.6, 4.5 Hz, 1H, *H*-7a'), 4.30–4.2 (m, 3H, *H*-5', 2 x *H*-9'), 4.09 (dd, *J* = 11.6, 7.6 Hz, 1H, *H*-7b'), 3.85 (ddd, *J* = 7.6, 5.7, 4.6 Hz, 1H, *H*-6'), 3.79 (s, 3H, OCH_3), 2.30 (dt, *J* = 12.7, 1.5 Hz, 1H, *H*-4a'), 2.13 (s, 3H, -COCH_3), 2.12 (s, 3H, -COCH_3), 2.11 – 2.06 (m, 1H, *H*-4b'). ^{13}C NMR (125 MHz, $CDCl_3$) δ 170.53 (*C*O), 169.87 (*C*O), 154.24, 152.63, 137.45, 128.49, 127.93, 127.39, 115.63, 114.69 (Ar), 101.02 (*C*-1'), 80.17 (*C*-2'), 79.83 (*C*-8'), 75.73 (*C*-3'), 68.72 (O*C*H_2Ph), 67.44 (*C*-5'), 66.06 (*C*-7'), 63.41 (*C*-9'), 63.33 (*C*-6'), 55.72 (OCH_3), 31.91 (*C*-4'), 20.84 (*C*H_3), 20.72 (*C*H_3). HRMS (ESI-TOF) *m*/*z*: $[M+Na]^+$ Calcd for $C_{27}H_{31}N_3NaO_9$ 564.1958; Found 564.1959.

(*S*)-Methyl-2-azido-2-((1*R*,3*S*,5*S*,6*R*,8*R*)-5-(benzyloxy)-8-hydroxy-6-((4-methoxyphenoxy)-methyl)-2,7-dioxabicyclo[3.2.1]octan-3-yl)acetate (15)

To an ice cooled solution of **12** (0.20 g, 0.43 mmol) in acetonitrile–water 1:1 (4 mL) was added bis-acetoxyiodobenzene (BAIB) (0.31g, 0.96 mmol) followed by $NaHCO_3$ (0.055 g, 0.65 mg)

and TEMPO (0.01 g, 0.0654 mmol). After stirring the reaction mixture for 3 h at 0 °C, NaS_2O_3 was added and solvent evaporated at reduced pressure. The residue was first extracted with diethyl ether (2 × 5 mL), and was discarded. The remaining residue was then extracted with ethyl acetate (3 × 5mL), washed with cold water, dried and concentrated to afford acid (0.176 g, 85.39%) enough pure to use in next step directly. IR (Neat) 3600–3000 (broad), 2120 and 1721 cm^{-1}; ^1H NMR (400 MHz, $CDCl_3$) δ 7.48 – 7.30 (m, 5H, Ar), 6.94 – 6.76 (m, 4H, Ar), 5.36 (s, 1H, *H*-1), 4.75 (dt, *J* = 10.7, 4.4 Hz, 1H, *H*-5'), 4.68 (d, *J* = 11.0 Hz, 1H, -OCH_2Ph), 4.64 – 4.59 (m, 1H, *H*-8'), 4.57 (d, *J* = 11.0 Hz, 1H, -OCH_2Ph), 4.35 – 4.24 (m, 4H, on D_2O exchange appeared as multiplate for 3H, *H*-6', 2 x *H*-9', O*H*), 3.93 (s, 1H, *H*-2'), 3.77 (s, 3H, -OCH_3), 2.37 (t, *J* = 12.5 Hz, 1H, *H*-4a'), 2.14 (s, 1H, exchanges with D_2O, O*H*), 2.04 (dd, *J* = 12.9, 4.8 Hz, 1H, *H*-4b'). ^{13}C NMR (100 MHz, $CDCl_3$) δ 170.72 (*C*O), 154.23, 152.61, 137.50, 137.42, 130.23, 128.69, 128.20, 127.56, 115.72, 114.76 (Ar), 102.49 (*C*-1'), 80.81 (*C*-2'), 79.36 (*C*-5'), 76.16 (*C*-3'), 69.94 (*C*-8'), 67.05 (O*C*H_2Ph), 66.28 (*C*-9'), 64.63 (*C*-6'), 55.75 (O*C*H_3), 29.86 (*C*-4'). HRMS (ESI-TOF) *m/z*: $[M+Na]^+$ Calcd for $C_{25}H_{27}N_3NaO_8$ 494.1534; Found 494.1528.

A solution of KOH (1.0 g) in ethanol (4 mL), in two neck round bottom flask equipped with addition funnel and U-shaped distillation condenser, was kept at 45 °C in fuming hood and then a solution of *N*-methyl-*N'*-nitro-*N*-nitrosoguanidine (MNNG, 0.5 g) in ether (10 mL) was added slowly. On complete addition, the solution turned bright yellow. The ether containing diazomethane was distilled off with the help of U-condenser till the disappearance of yellow colour of distillate. The diazomethane thus prepared was added to a stirred solution of crude acid (0.176 g), as prepared above, in diethyl ether (2 mL) and stirring continued at room temperature for 6 h. Excess of CH_2N_2 was removed by bubbling nitrogen into the reaction mixture for 15 min and solvent was removed under vacuum to yield crude product. Purification by column

chromatography (*n*-hexane/EtOAc = 3:2) provided methyl ester **15** (0.17 g, 95.9%) as oil: R_f 0.55 (*n*-hexane/ethyl acetate = 7/3); $[\alpha]_D^{25}$ 18.27 (*c* 1.05, $CHCl_3$); IR (Neat) 3600–3200 (broad), 2120 and 1739 cm^{-1}; ^1H NMR (500 MHz, $CDCl_3$) δ 7.41 – 7.30 (m, 5H, Ar), 6.92 – 6.85 (m, 2H, Ar), 6.87 – 6.81 (m, 2H, Ar), 5.34 (s, 1H, *H*-1'), 4.74 (dt, *J* = 10.7, 4.5 Hz, 1H, *H*-5'), 4.69 (d, *J* = 11.0 Hz, 1H, -OCH_2Ph), 4.62 (dd, *J* = 5.1, 3.6 Hz, 1H, *H*-8'), 4.58 (d, *J* = 11.0 Hz, 1H, -OCH_2Ph), 4.36 – 4.26 (m, 3H, *H*-6', 2 x *H*-9'), 3.93 (s, 1H, *H*-2'), 3.82 (s, 3H, OCH_3), 3.78 (s, 3H, OCH_3), 2.58 (s, 1H, exchanges with D_2O, OH), 2.32 (dt, *J* = 12.6, 1.4 Hz,1H, *H*-4a'), 1.99 (dd, *J* = 12.6, 4.8 Hz, 1H, *H*-4e'). ^{13}C NMR (125 MHz, $CDCl_3$) δ 168.01 (*C*O), 154.18, 152.59, 137.49, 128.67, 128.15, 127.55, 115.66, 114.68 (Ar), 102.61(*C*-1'), 80.75 (*C*-2'), 79.29 (*C*-5'), 76.03 (*C*-3'), 69.73 (*C*-7'), 66.99 (O*C*H_2Ph), 66.25 (*C*-8'), 64.74 (*C*-6'), 55.73 (O*C*H_3), 53.07 (COO*C*H_3), 29.77(*C*-4'). HRMS (ESI-TOF) *m*/*z*: $[M+Na]^+$ Calcd for $C_{24}H_{27}N_3NaO_8$ 508.1696; Found 508.1705.

(*S*)-Methyl 2-((1*R*,3*S*,5*R*,6*R*,8*R*)-8-acetoxy-5-(benzyloxy)-6-((4-methoxyphenoxy)methyl) -2,7-dioxabicyclo[3.2.1]octan-3-yl)-2-azidoacetate (16)

To an ice cooled solution of **15** (0.15 g, 0.31 mmol) in pyridine (1 mL) was added Ac_2O (44 μL, 0.46 mmol) and mixture was stirred for 2 h at room temperature. Chloroform (10 mL) was added, organic phase was washed with 10% HCl (2 x 05 mL) followed by saturated $NaHCO_3$ solution (5 mL) and brine. The organic layer was dried and evaporated under reduced pressure and residue thus obtained was purified by column chromatography (*n*-hexanes/ EtOAc = 9:1) to yield **16** (0.155 g, 95.1%) as viscous oil: R_f 0.5 (*n*-hexane/ethyl acetate = 4/1); $[\alpha]_D^{25}$ 21.83 (*c* 0.85, $CHCl_3$); IR (Neat) 2121 and 1740 cm^{-1}; ^1H NMR (500 MHz, $CDCl_3$) δ 7.40 – 7.29 (m, 5H, Ar), 7.00 – 6.80 (m, 4H, Ar), 5.41 (s, 1H, *H*-1'), 5.09 (s, 1H, *H*-2'), 4.75 (dt, *J* = 10.7, 4.4 Hz,

1H, *H*-5’), 4.63 – 4.60 (m, 2H, -OCH_2Ph, *H*-8’), 4.51 (d, *J* = 10.7 Hz, 1H, -OCH_2Ph), 4.33 (d, *J* = 4.1 Hz, 1H, *H*-6’), 4.29 (m, 2H, *H*-9’), 3.84 (s, 3H, COOCH_3), 3.79 (s, 3H, OCH_3), 2. 45 (dt, *J* = 12.5, 1.4 Hz, 1H, *H*-4a’), 2.12 (s, 3H, COCH_3), 1.98 (dd, *J* = 12.5, 4.7 Hz, 1H, *H*-4e’). ^{13}C NMR (125 MHz, $CDCl_3$) δ 169.79 (O*C*O), 167.96 (O*C*O), 154.20, 152.60, 137.41, 128.44, 127.88, 127.36, 127.30, 115.60, 115.50, 114.65 (Ar), 101.12 (*C*-1’), 80.16 (*C*-2’), 79.69 (*C*-5’), 75.55 (*C*-3’), 69.54 (*C*-7’), 67.41 (O*C*H_2Ph), 65.96 (*C*-8’), 64.66 (*C*-6’), 55.72 (COO*C*H_3), 53.08 (O*C*H_3), 30.40 (C-4’), 20.84 (CO*C*H_3). HRMS (ESI-TOF) *m*/*z*: $[M+Na]^+$ Calcd for $C_{26}H_{29}N_3NaO_9$ 550.1801; Found 550.1786.

(*S*)-Methyl 2-(((benzyloxy)carbonyl)amino)-2-((1*R*,3*S*,5*S*,6*R*,8*R*)-5,8-dihydroxy-6-((4-methoxyphenoxy)methyl)-2,7-dioxabicyclo[3.2.1]octan-3-yl)acetate (19)

To a stirred solution of **15** (0.20 g, 0.41 mmol) and 10% Pd/C (0.02 g) in methanol (5 mL) at 25 °C was added triethylsilane (0.65 mL, 4.12 mmol) drop wise. The reaction mixture was stirred till complete consumption of starting material (4 h). The reaction mixture was filtered over celite, the residue washed with methanol (5 mL) and the filtrate evaporated at reduced pressure to get the crude amine (152 mg). The crude amine thus obtained in the above step was dissolved in methanol–water (9:1, 5 mL) to which $NaHCO_3$ (85 mg, 1.02 mmol) was added and the reaction mixture was cooled to 0 °C. To this ice cooled solution, CbzCl (carbobenzyloxy chloride) (0.174 mL, 0.61 mmol) was added dropwise and reaction mixture was stirred for 3 h while allowing to reach rt. After completion of reaction, the solvent was concentrated and the resulting crude mixture was dissolved in ethyl acetate (10 mL) and washed with water. The organic phase was dried, the solvent evaporated and the residue was purified using column chromatography (*n*-hexanes/ EtOAc = 3:1) to provide **19** (0.185 g, 90.5%) as viscous oil: R_f 0.5 (*n*-hexane/ethyl acetate = 3/7); $[\alpha]_D^{25}$ 45.10 (*c* 0.61, $CHCl_3$); IR (Neat) 1691 and 1740 cm^{-1}; ^{1}H NMR (500 MHz,

$CDCl_3$) δ 7.41 – 7.14 (m, 5H, Ar), 6.84 (d, *J* = 9.0 Hz, 2H, Ar), 6.78 (d, *J* = 9.0 Hz, 2H, Ar), 5.86 (d, *J* = 8.4 Hz, 1H, N*H*), 5.19 (s, 1H, *H*-1'), 5.11 – 4.99 (m, 2H, OCH_2Ph), 4.53 – 4.42 (m, 2H, *H*-6, *H*-8'), 4.40 – 4.28 (m, 1H, *H*-5'), 4.20 (d, *J* = 4.4 Hz, 2H, *H*-9'), 3.94 (bs, 1H, exchanges with D_2O, O*H*), 3.71 (s, 3H, COOCH_3), 3.69 (s, 3H, OCH_3), 3.59 (s, 1H, *H*-2'), 2.49 (bs, 1H, exchanges with D_2O, O*H*), 2.15 (t, *J* = 12.3 Hz, 1H, *H*-4a'), 2.05 (dd, *J* = 12.3, 4.5 Hz, 1H, *H*-4e'). ^{13}C NMR (125 MHz, $CDCl_3$) δ 169.94 (O*C*O), 156.00 (N*C*O), 154.18, 152.50, 135.97, 128.54, 128.25, 128.12, 115.67, 114.66, 102.03 (*C*-1'), 78.66 (*C*-2'), 78.31 (*C*-5'), 75.97 (*C*-8'), 70.78 (*C*-3'), 67.28 (O*C*H_2Ph), 66.17 (*C*-9'), 56.94 (*C*-6'), 55.69 (O*C*H_3), 52.62 (COO*C*H_3), 34.71 (*C*-4'). HRMS (ESI-TOF) *m*/*z*: $[M+Na]^+$ Calcd for $C_{26}H_{31}NNaO_9$ 524.1897; Found 524.1903.

(1*R*,3*S*,5*R*,6*R*,8*R*)-6-Acetoxymethyl-3-((*S*)-1-(((benzyloxy)carbonyl)amino)-2-methoxy-2-oxoethyl)-2,7-dioxabicyclo[3.2.1]octane-5,8-diyl diacetate (20)

Ceric ammonium nitrate (0.37 mg, 0.67 mmol) was added to an ice cooled solution of **19** (0.17 g, 0.34 mmol) in acetonitrile–water (4:1, 4 mL) and stirred for 0.5 h. To the reaction mixture, a saturated solution of $NaHCO_3$ (2 mL) was added and the solvent evaporated. The resulting crude aqueous layer was extracted with ethyl acetate (2 × 10 mL), organic phase was dried and evaporated to get crude product (0.13 g), which was utilized as it was for the next step. To the crude compound was added pyridine (1.5 mL) followed by Ac_2O (0.14 mL, 1.3 mmol) and the reaction mixture was stirred for 10 h at rt. After completion of reaction, dichloromethane (15 mL) was added and the resulted solution was washed with 10% HCl (2 × 05 mL). The organic phase was dried, evaporated to get a residue that was purified using column chromatography to get **20** (0.135 g, 76.4%) as thick liquid: R_f 0.45 (*n*-hexane/ethyl acetate = 1/1); $[\alpha]_D^{25}$ 50.62 (*c* 0.57, $CHCl_3$); IR (Neat) 1694 and 1742 cm^{-1}; ^{1}H NMR (500 MHz, $CDCl_3$) δ 7.40 – 7.30 (m, 5H,

Ar), 5.74 (d, J = 8.4 Hz, 1H, NH), 5.35 (s, 1H, H-1'), 5.34 (s, 1H, H-2'), 5.12 (ABq, J = 12.2 Hz, 2H, OCH_2Ph), 4.54 (d, J = 11.8 Hz, 1H, H-9a'), 4.47 (d, J = 8.5 Hz, 2H, H-6', H-8'), 4.41 – 4.24 (m, 2H, H-9b', H-5'), 3.82 (s, 3H, COOCH_3), 3.04 (t, J = 12.4 Hz, 1H, H-4a'), 2.13 (s, 3H, OCCH_3), 2.09-2.04 (m, 7H, H-4e, 2 x OCCH_3). ^{13}C NMR (126 MHz, $CDCl_3$) δ 170.68 (O*C*O), 169.30 (O*C*O), 169.21 (O*C*O), 168.84 (O*C*O), 155.74 (N*C*O), 135.99, 128.53, 128.23, 128.14, 100.67 (*C*-1'), 79.14 (*C*-2'), 78.82 (*C*-5'), 76.74 (*C*-8'), 70.58 (*C*-3'), 67.26 (OCH$_2$Ph), 61.41 (*C*-9'), 56.56 (*C*-6'), 52.75 (OCH_3), 31.58 (*C*-4'), 21.20 (OCCH_3), 20.77 (OCCH_3), 20.69 (OCCH_3). HRMS (ESI-TOF) *m*/*z*: $[M+Na]^+$ Calcd for $C_{24}H_{29}NNaO_{12}$ 546.1582; Found 546.1585.

(2*S*,3*R*,4*R*,6*S*)-4-((*R*)-1-Acetoxy-2-(4-methoxyphenoxy)ethyl)-6-((*S*)-1-azido-2-methoxy-2-oxoethyl)-4-(benzyloxy)tetrahydro-2*H*-pyran-2,3-diyl diacetate (21)

Compound **16** (0.05 g, 0.095 mmol) in Ac_2O (0.5 mL) was cooled to 0 °C and to this cold solution cat. H_2SO_4 (3–5 μL) was added and stirred for 30 min at this temperature. Saturated solution of $NaHCO_3$ was added and extracted with ethyl acetate (2 x 5 mL). The organic phase was dried, evaporated to get a residue which was purified by column chromatography (*n*-hexanes/ EtOAc = 9:1) to give **21** (0.051 g, 83.79%) as oil: R_f 0.4 (*n*-hexane/ethyl acetate = 4/1); $[\alpha]_D^{25}$ 30.22 (*c* 2.0, $CHCl_3$); IR (Neat) 1745, 2120 cm^{-1}; The ^{1}H NMR spectra showed a mixture of α:β anomers in the ratio 1:1.5, The ^{1}H NMR (300 MHz, $CDCl_3$) of major isomer δ 7.50 – 7.18 (m, 5H), 6.96 – 6.67 (m, 4H), 6.38 (d, J = 6.9 Hz, 1H) and 5.60 (m, 1H), 6.28 (d, J = 3.7 Hz) and 5.45 (d, J = 3.9 Hz, 1H), 6.11 (d, J = 7.6 Hz) and 5.35 (d, J = 7.5 Hz, 1H), 4.78 – 4.03 (m, 6H), 3.97 – 3.62 (m, 6H), 2.45–1.95 (m, 11H). ^{13}C NMR (126 MHz, $CDCl_3$) δ 170.41, 170.38, 169.20, 169.07, 169.06, 154.08, 152.93, 152.56, 137.88, 137.51, 128.46, 127.75, 127.62, 127.47, 126.84, 115.99, 115.58, 114.71, 114.62, 91.66, 89.68, 78.41, 77.32, 77.29, 77.03, 76.78, 73.40,

72.69, 71.04, 70.38, 70.30, 69.28, 68.08, 67.05, 63.63, 63.01, 62.52, 55.73, 52.53, 31.65, 21.13, 20.94, 20.88, 20.81, 20.65, 20.60. HRMS (ESI-TOF) *m*/*z*: $[M+Na]^+$ Calcd for $C_{30}H_{35}N_3NaO_{12}$ 652.2118; Found 652.2115.

Preparation of pyranosyl nucleoside 2

To a solution of thymine (0.0065 g, 0.051 mmol) in dry dichloroethane (2 mL) at rt was added *N,O*-bis(trimethylsilyl)acetamide (0.035 mL, 0.14 mmol). The reaction mixture was stirred for 20 min at 50 °C till to get clear solution and then cooled to 0 °C. To this, a solution of **21** (0.03 g, 0.047 mmol) in dry dichloroethane (1 mL) was added followed by dropwise addition of trimethylsilyl trifluoromethane sulfonate (0.013 mL, 0.070 mmol). The resulting solution was stirred for 15 h at 50 °C and then cooled to 0 °C. Chloroform (5 mL) and then saturated aq $NaHCO_3$ solution (5 mL) was added. The reaction mixture was extracted with chloroform (2 × 5 mL) and the combined organic layer was washed with brine. Organic phase was then dried over anhydrous sodium sulfate, filtered off and concentrated to get the crude product. Purification by column chromatography using first *n*-hexane/ethyl acetate = 9/1 gave starting compound **21** (0.011 g, 30%). Further elution with (*n*-hexane/ethyl acetate = 1/1) gave nucleoside **2** (0.013 g, 42.1%) as semisolid. R_f 0.56 (*n*-hexane/ethyl acetate = 1/9); $[\alpha]_D^{25}$ 16.87 (*c* 0.4, $CHCl_3$); IR (Neat) 3300 (br), 1745 cm^{-1}; ^1H NMR (500 MHz,) δ 8.42 (s, 1H, N*H*), 7.40 – 7.24 (m, 6H, Ar), 6.83–6.82 (m, 4H, Ar), 6.33 (d, *J* = 9.3 Hz, 1H, *H*-1’), 5.72 (apparent t , *J* = 4.0 Hz, 1H, *H*-8’), 5.32 (d, *J* = 9.3 Hz, 1H, *H*-2’), 4.71 (m, *J* = 13.0 Hz, 1H, *H*-5’), 4.63 (d, *J* = 10.4 Hz, 1H, -OCH_2Ph), 4.57 (d, *J* = 10.4 Hz, 1H, -OCH_2Ph), 4.43 (d, *J* = 3.5 Hz, 1H, *H*-6’), 4.37 (dd, *J* = 10.9, 3.3 Hz, 1H, *H*-9a’), 4.27 (dd, *J* = 10.9, 5.5 Hz, 1H, *H*-9b’), 3.78 (s, 6H, OCH_3, $COOCH_3$), 2.43 (d, *J* = 13.0 Hz, 1H, *H*-4a’), 2.30–2.25 (m, 4H, *H*-4b’, $COOCH_3$), 2.03 (s, 3H, $COOCH_3$),

1.98 (s, 3H, CH_3). ^{13}C NMR (125 MHz, $CDCl_3$) δ 169.47 (*C*OO and *C*ON), 167.25 (O*C*O), 163.07 (O*C*O), 154.30, 152.63 (Ty), 150.38, 137.09 (Ty), 134.59, 128.52, 127.92, 127.47, 127.31, 116.05, 115.92, 114.69 (Ar), 112.05 (Ty) 79.8 (*C*-1'), 78.08 (*C*-2'), 72.87 (*C*-5'), 72.05 (*C*-8'), 70.39 (*C*-3'), 67.53 (*C*-6'), 64.82 (O*C*H_2Ph), 64.75 (*C*-9'), 55.71 (*C*OO*C*H_3), 53.20 (O*C*H_3), 30.61 (*C*-4'), 21.14 (OCO*C*H_3), 20.48 (OCO*C*H_3), 12.54 (*C*H_3). HRMS (ESI-TOF) *m*/*z*: $[M+Na]^+$ Calcd for $C_{33}H_{37}N_5NaO_{12}$ 718.2331; Found 718.2329.

Conformational preferences of 1, 3 anhydrosugars 13 and 14 by Density Functional Theory (DFT) method:

To corroborate our experimental results obtained by ^1H NMR studies of synthesized 1,3-anhydrosugars **13** and **14**, and to predict their conformational preferences we have performed complete geometry optimizations by Density Functional Theory (DFT) method using B3LYP (6-31G** basis set). The molecular structures of 1, 3 anhydrosugars **13** and **14** were generated and optimized using Spartan-14 commercially available quantum chemical software. [1]

References:

1. Shao, Y.; Molnar, L. F.; Jung, Y.; *et al*. *Physical Chemistry Chemical Physics*, **2006**, *8*, 3172–3191.
