# Supplementary material for: Synthesis of the C8’-epimeric thymine pyranosyl amino acid core of amipurimycin
Source: Beilstein J Org Chem. 2016 Aug 5;12:1765–71. doi: 10.3762/bjoc.12.165 (PMC4979907; doi:10.3762/bjoc.12.165)
Supplement: File 2 — Copies of NMR spectra. [file Beilstein_J_Org_Chem-12-1765-s002.pdf]

# Supporting Information

**for**

# Synthesis of the C8’-epimeric thymine pyranosyl amino acid core of amipurimycin

Pramod R. Markad, Navanath Kumbhar and Dilip D. Dhavale*

Address: Garware Research Centre, Department of Chemistry, Savitribai Phule Pune University (Formerly University of Pune), Pune-411007, India.

Email: Dilip D. Dhavale - ddd@chem.unipune.ac.in

*Corresponding author

## Copies of NMR spectra

Table of Contents

$^{1}$H and $^{13}$C spectra of new compounds **4–21** and **2** **s2–s18**

NOESY spectrum of compound **14** **s19**

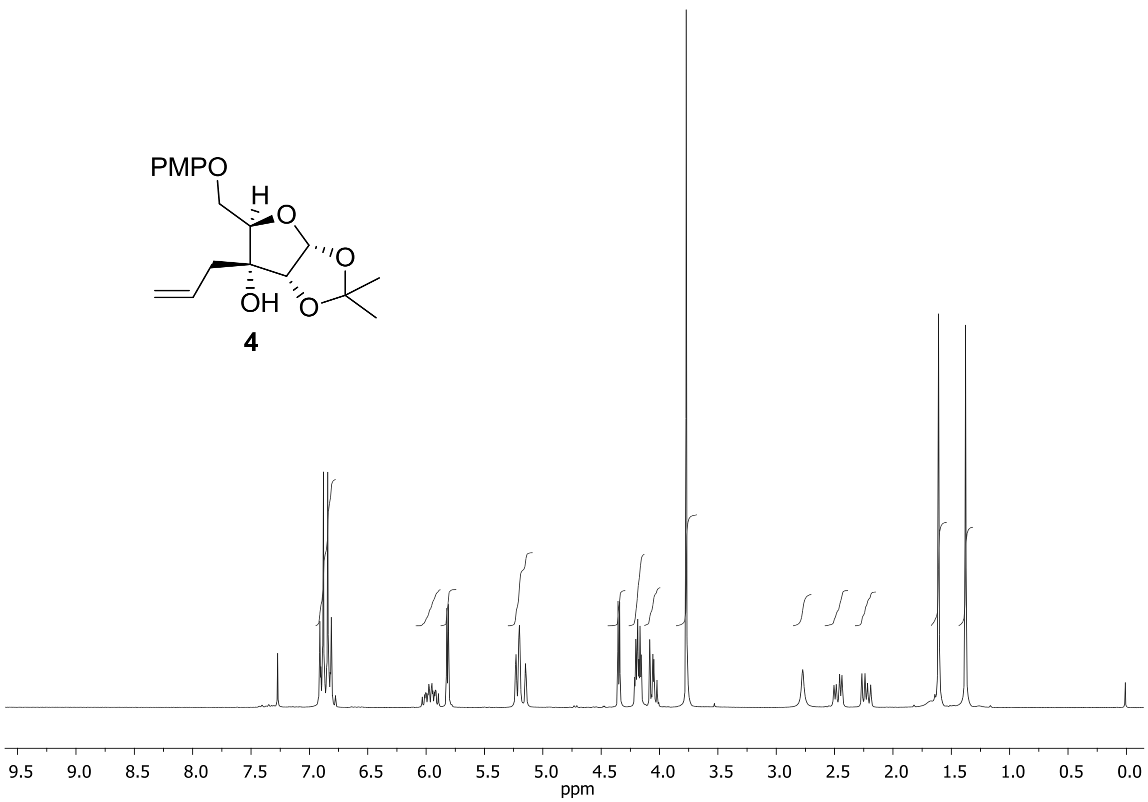


**Figure 1:** $^{1}$H NMR (300 MHz, $CDCl_3$) spectrum of compound **4**.

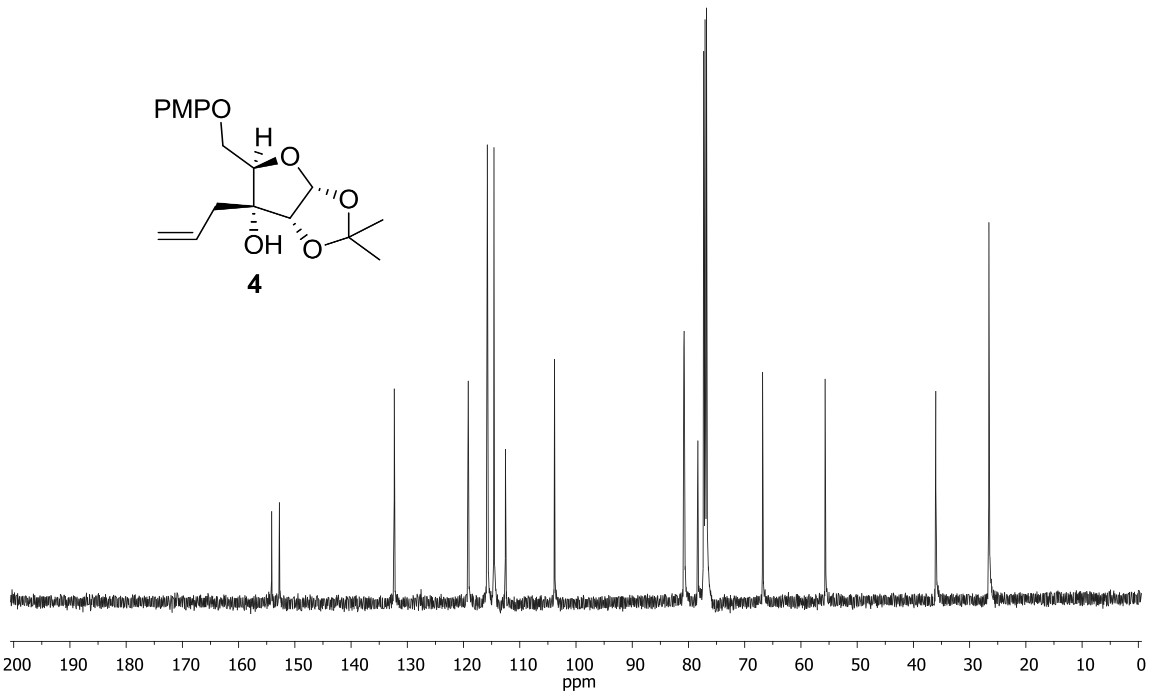


**Figure 2:** $^{13}$C NMR (125 MHz, $CDCl_3$) spectrum of compound **4**.

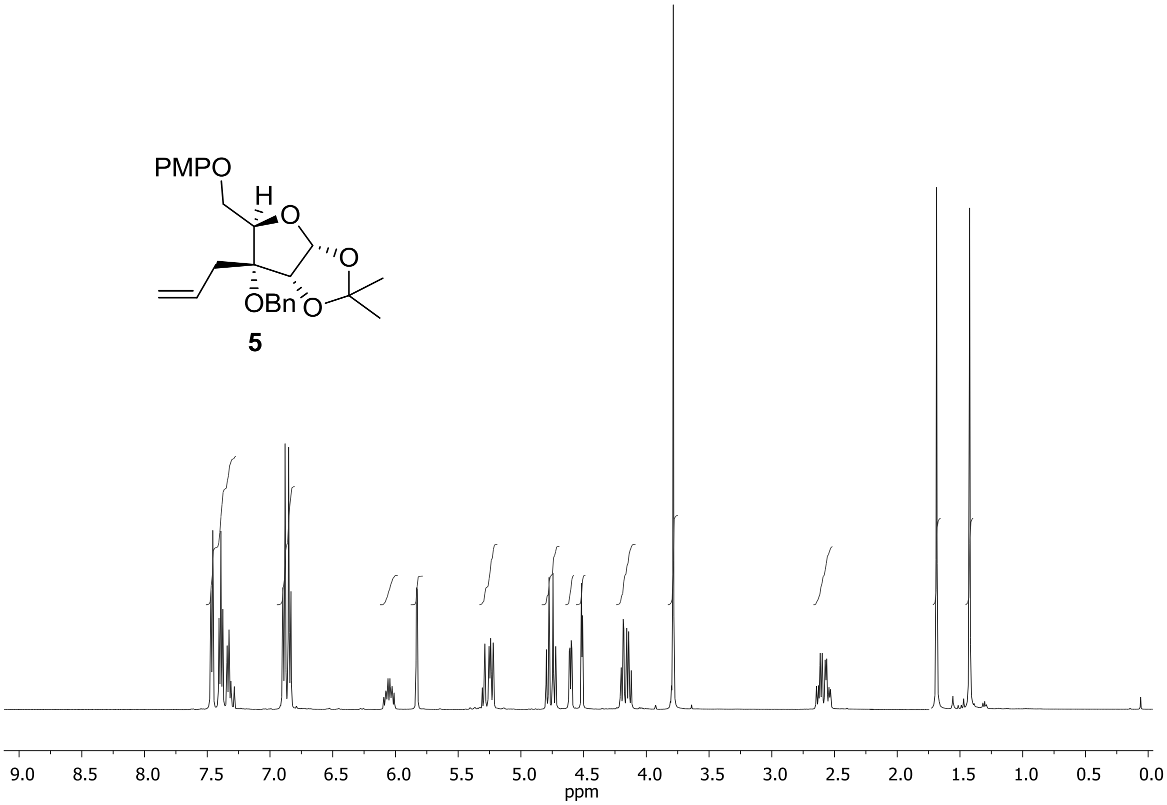


**Figure 3:** $^{1}$H NMR (500 MHz, $CDCl_3$) spectrum of compound **5**.

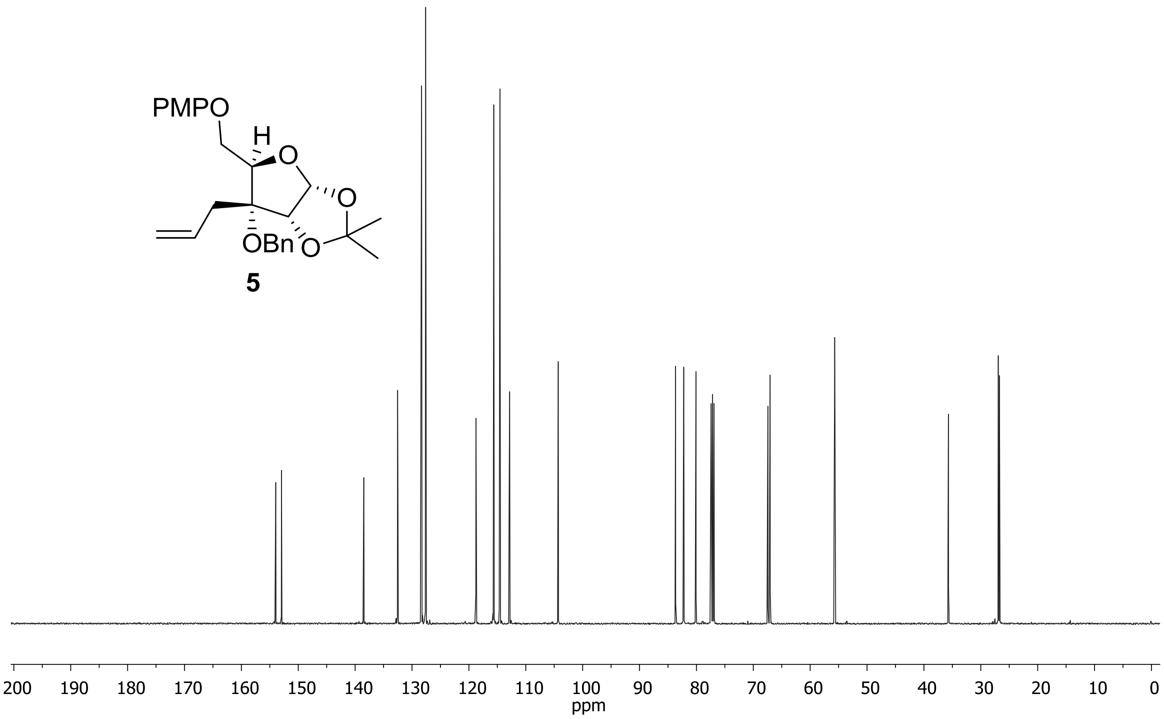


**Figure 4:** $^{13}$C NMR (125 MHz, $CDCl_3$) spectrum of compound **5**.

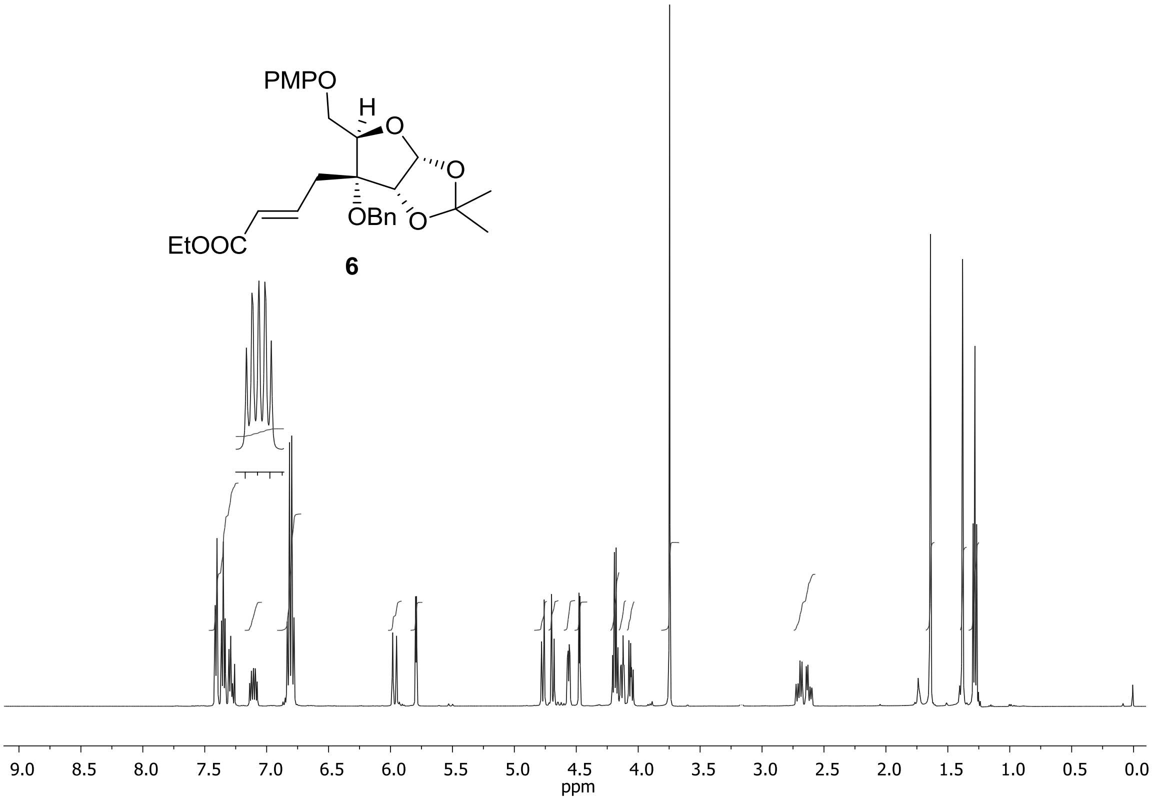


**Figure 5:** $^{1}H$ NMR (500 MHz, $CDCl_3$) spectrum of compound **6**

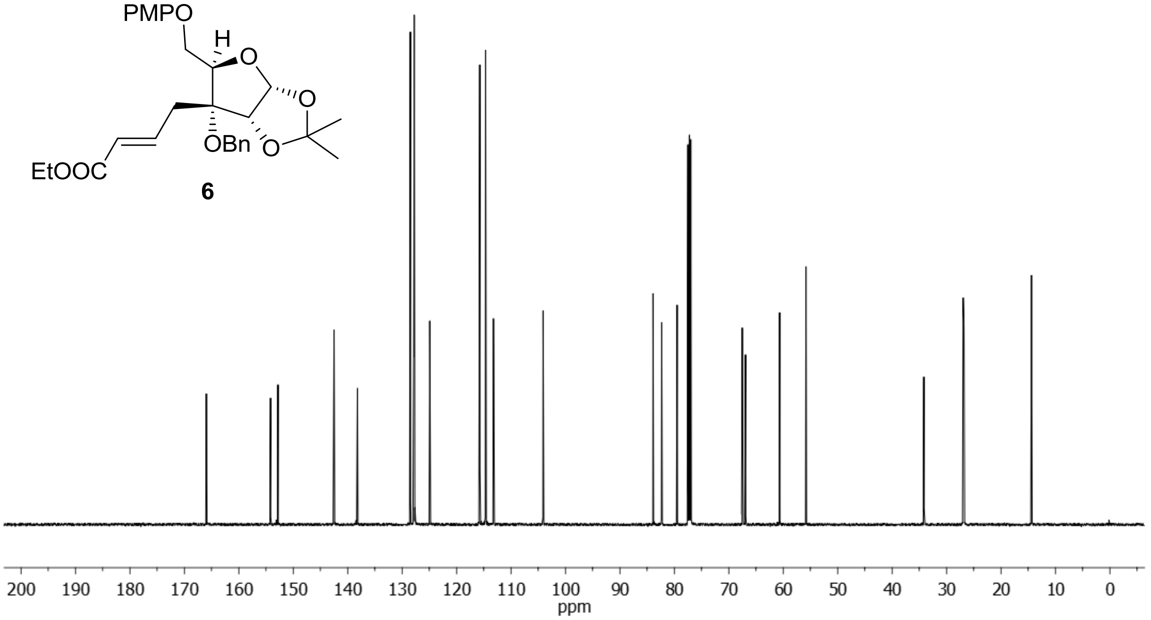


**Figure 6:** $^{13}C$ NMR (125 MHz, $CDCl_3$) spectrum of compound **6**

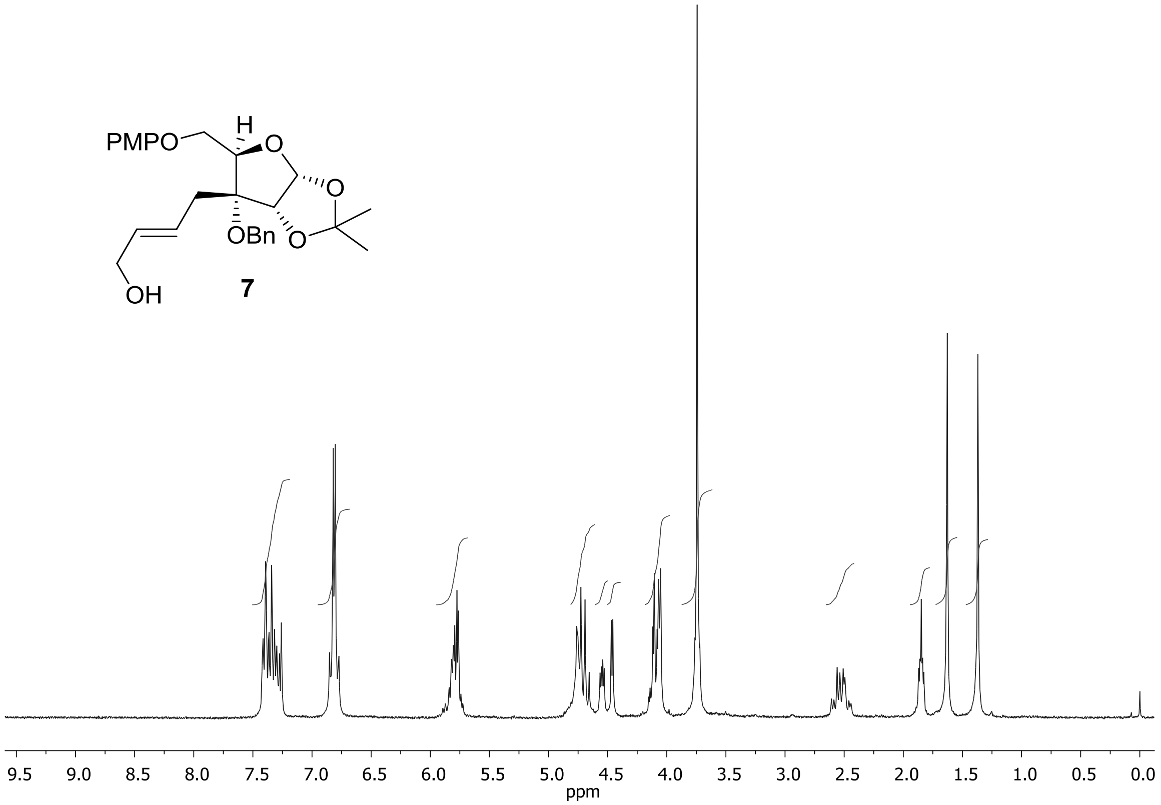


**Figure 7:** $^{1}$H NMR (300 MHz, $CDCl_3$) spectrum of compound **7**

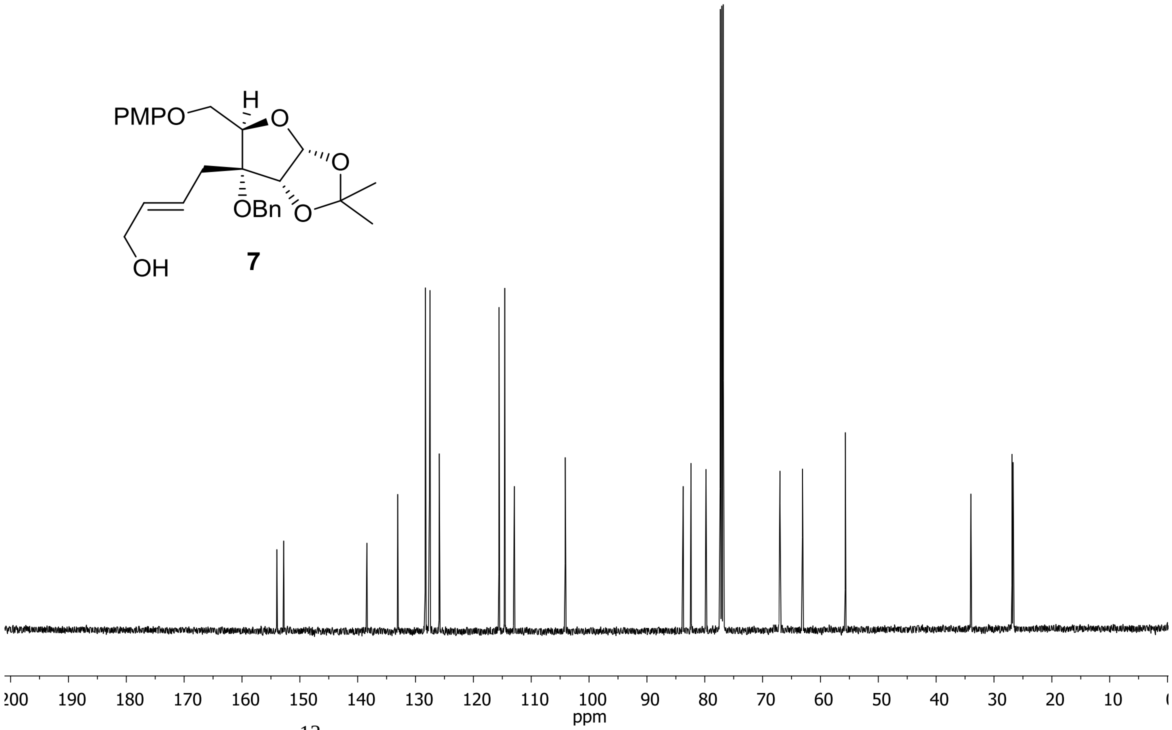


**Figure 8:** $^{13}$C NMR (125 MHz, $CDCl_3$) spectrum of compound **7**

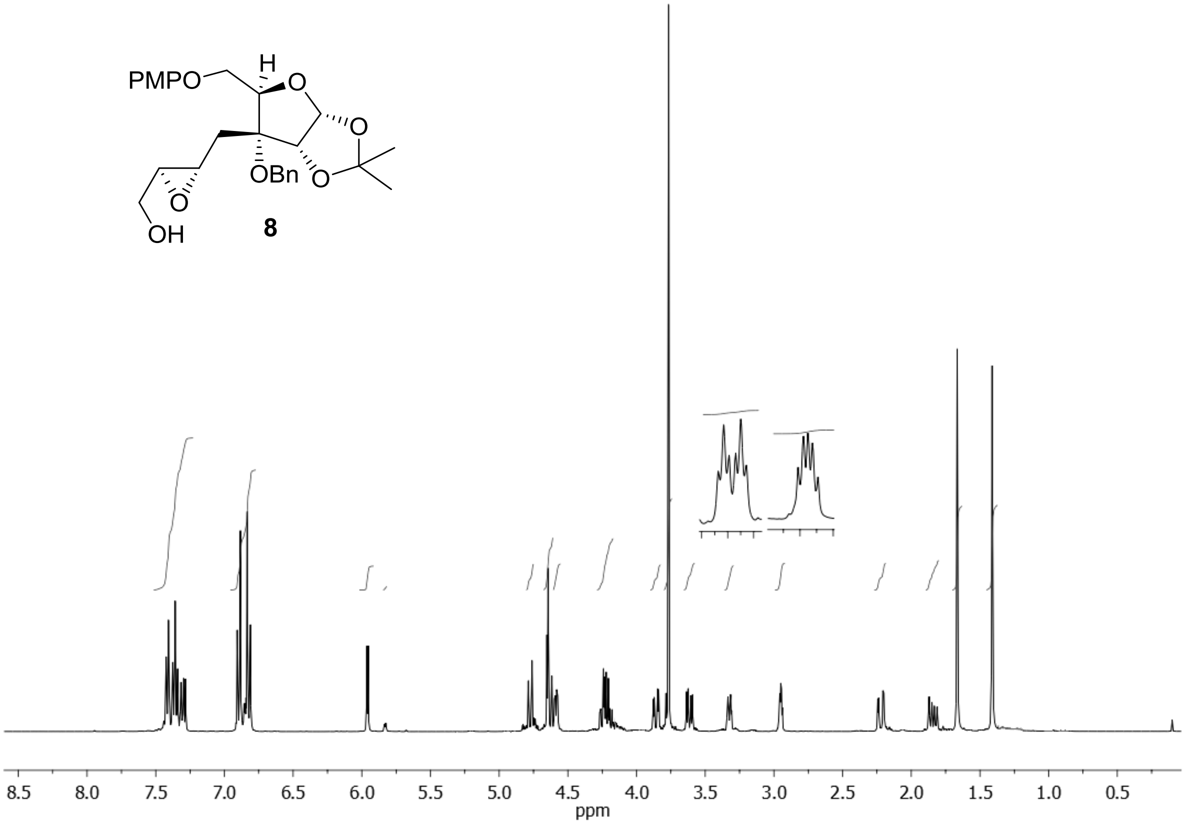


**Figure 9:** $^{1}H$ NMR (400 MHz, $CDCl_3$) spectrum of compound **8**

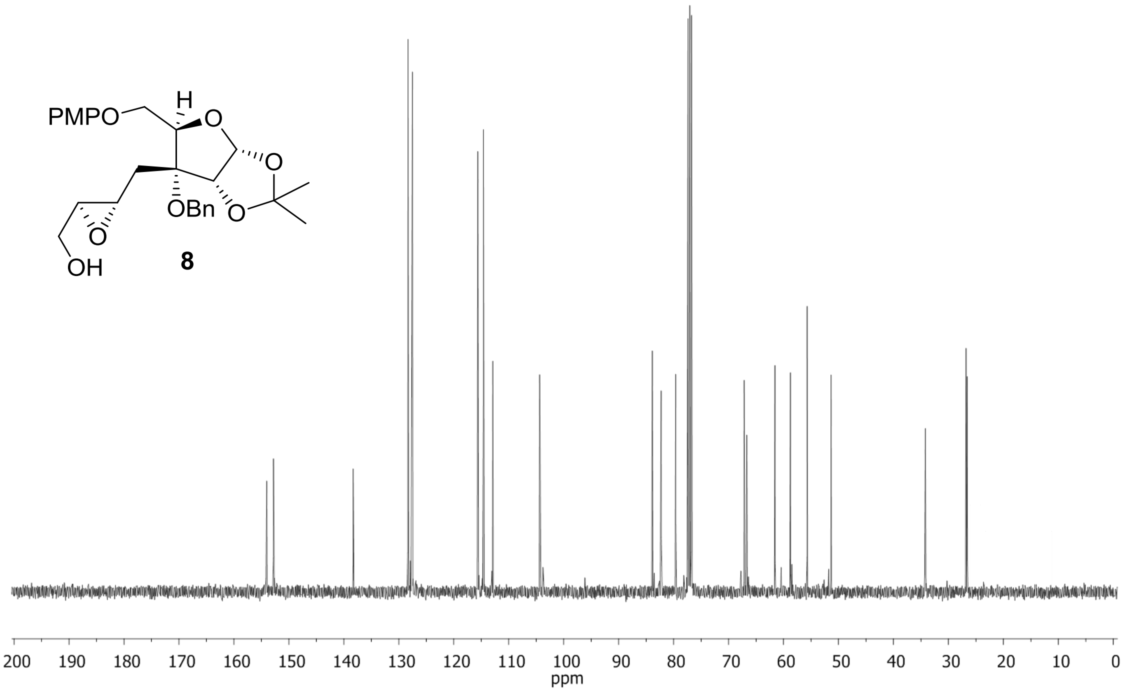


**Figure 10:** $^{13}C$ NMR (100 MHz, $CDCl_3$) spectrum of compound **8**

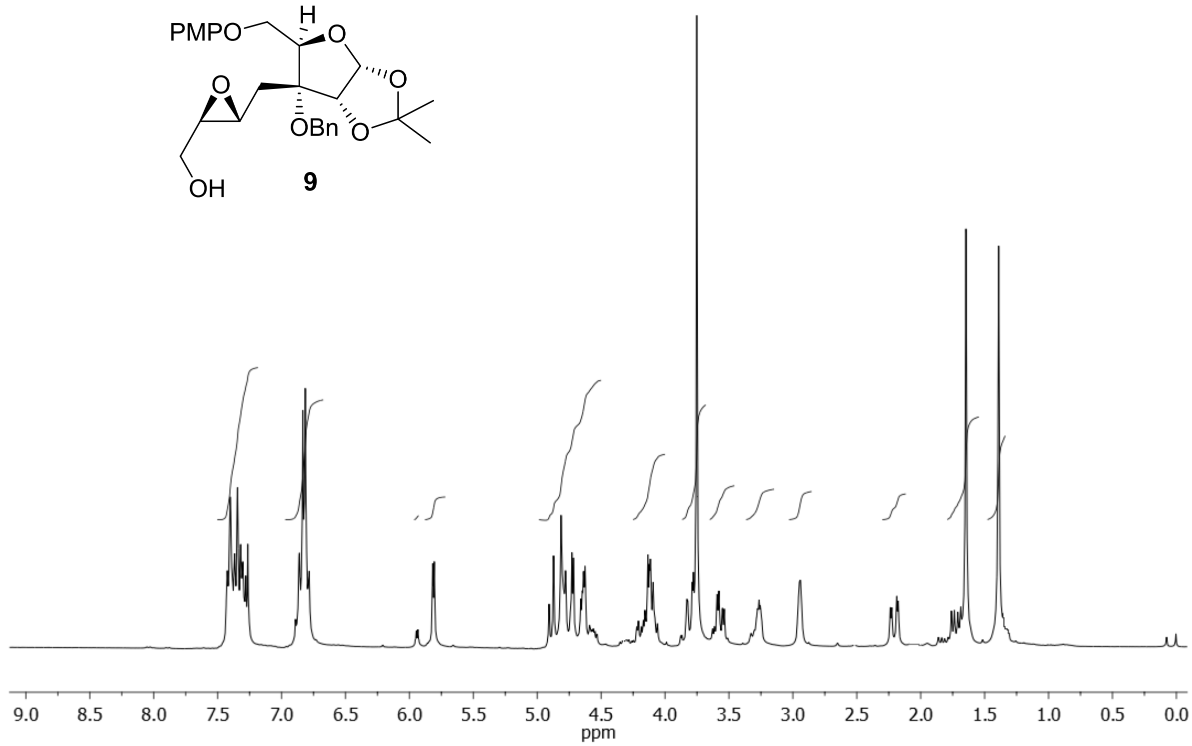


**Figure 11:** $^{1}H$ NMR (300 MHz, $CDCl_3$) spectrum of compound **9**

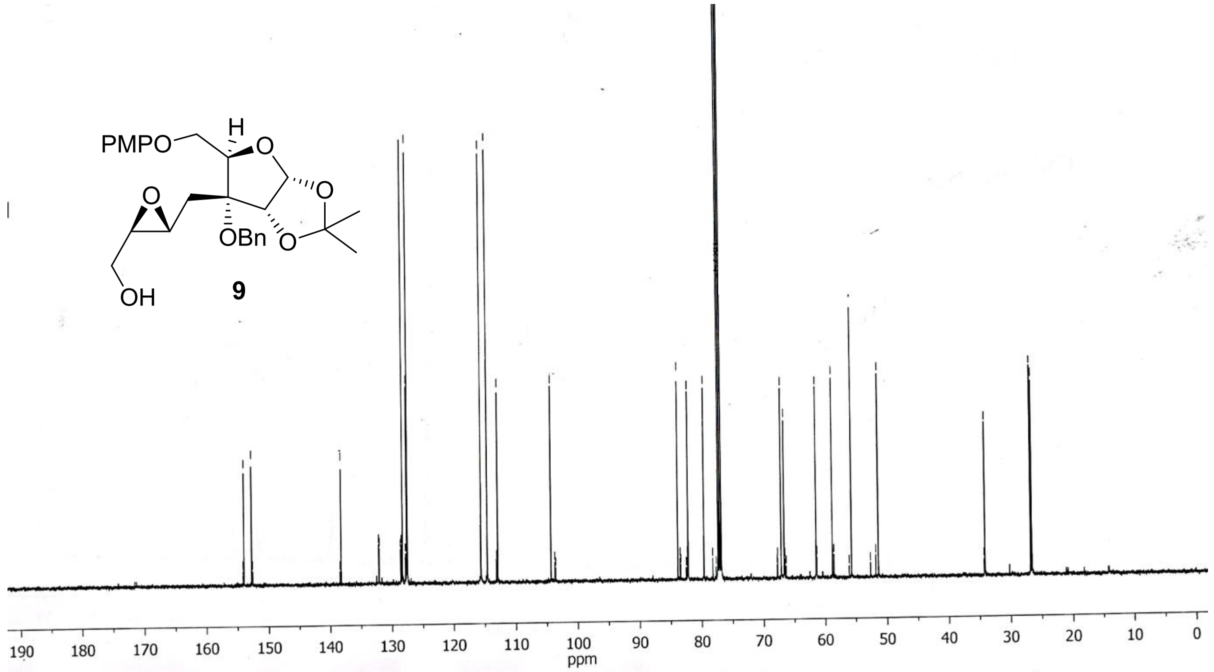


**Figure 12:** $^{13}C$ NMR (125 MHz, $CDCl_3$) spectrum of compound **9**

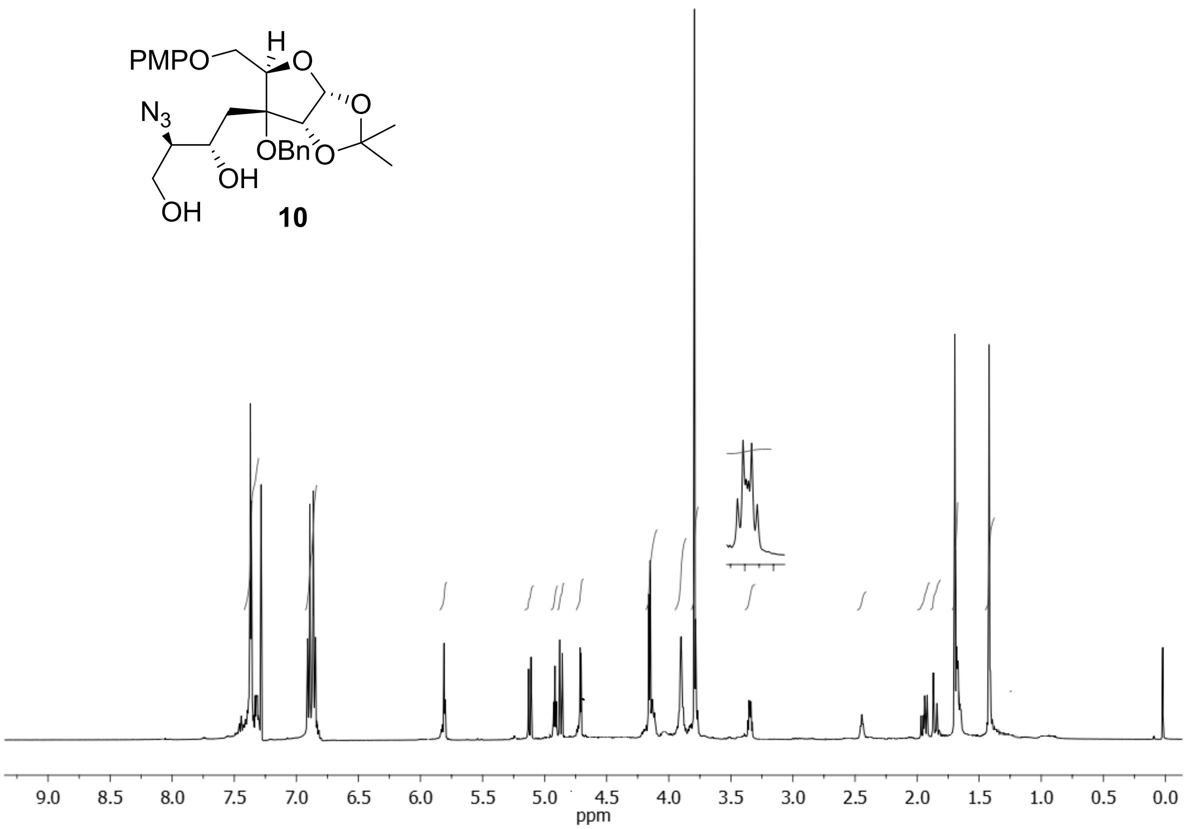


**Figure 13:** $^{1}$H NMR (500 MHz, $CDCl_3$) spectrum of compound **10**

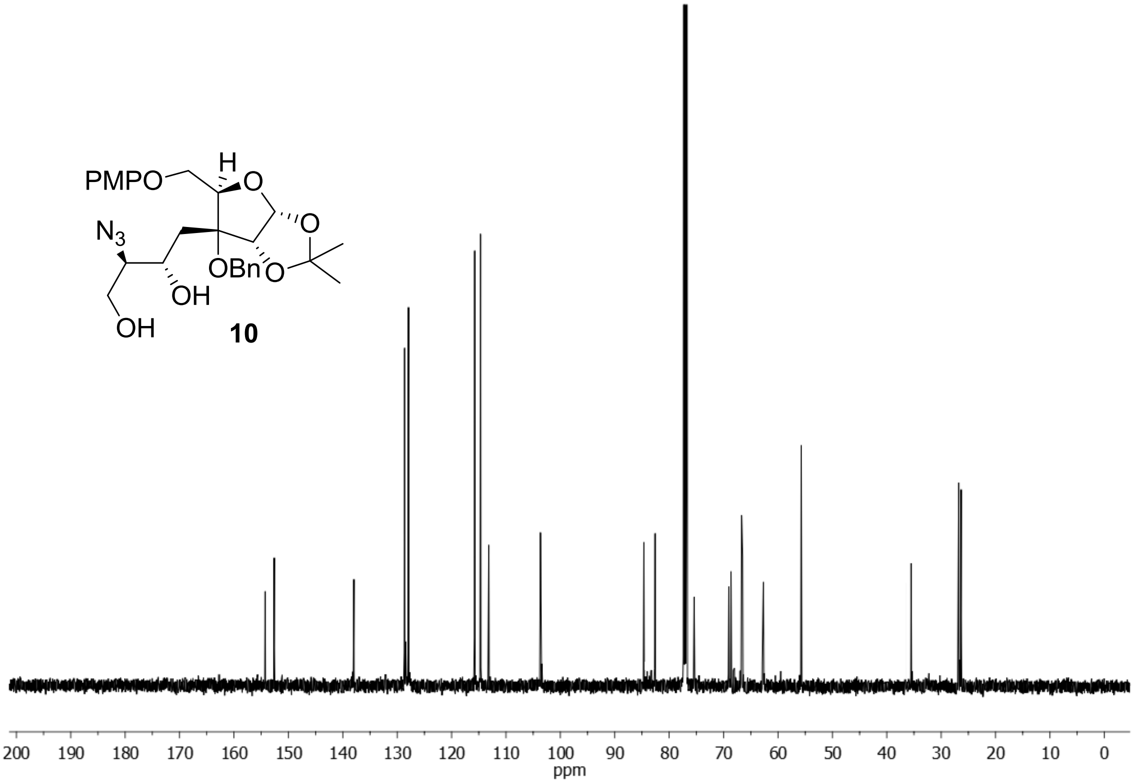


**Figure 14:** $^{13}$C NMR (125 MHz, $CDCl_3$) spectrum of compound **10**

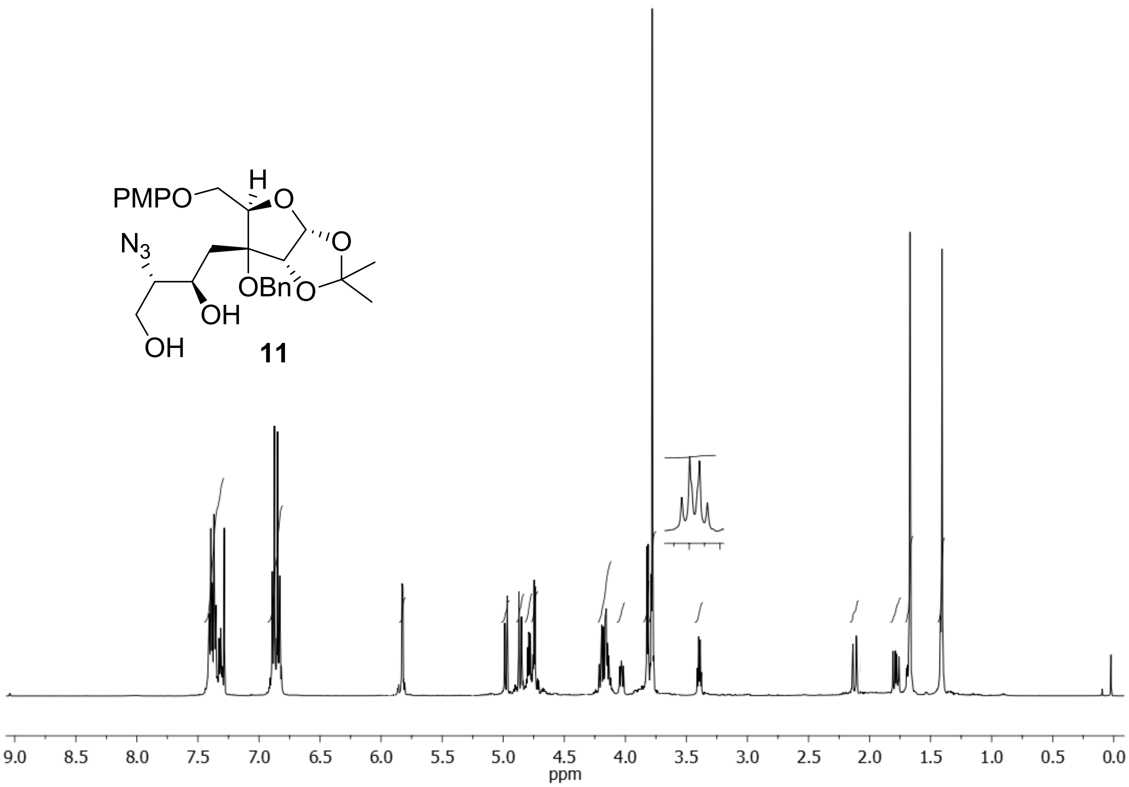


**Figure 15:** $^{1}$H NMR (500 MHz, $CDCl_3$) spectrum of compound **11**

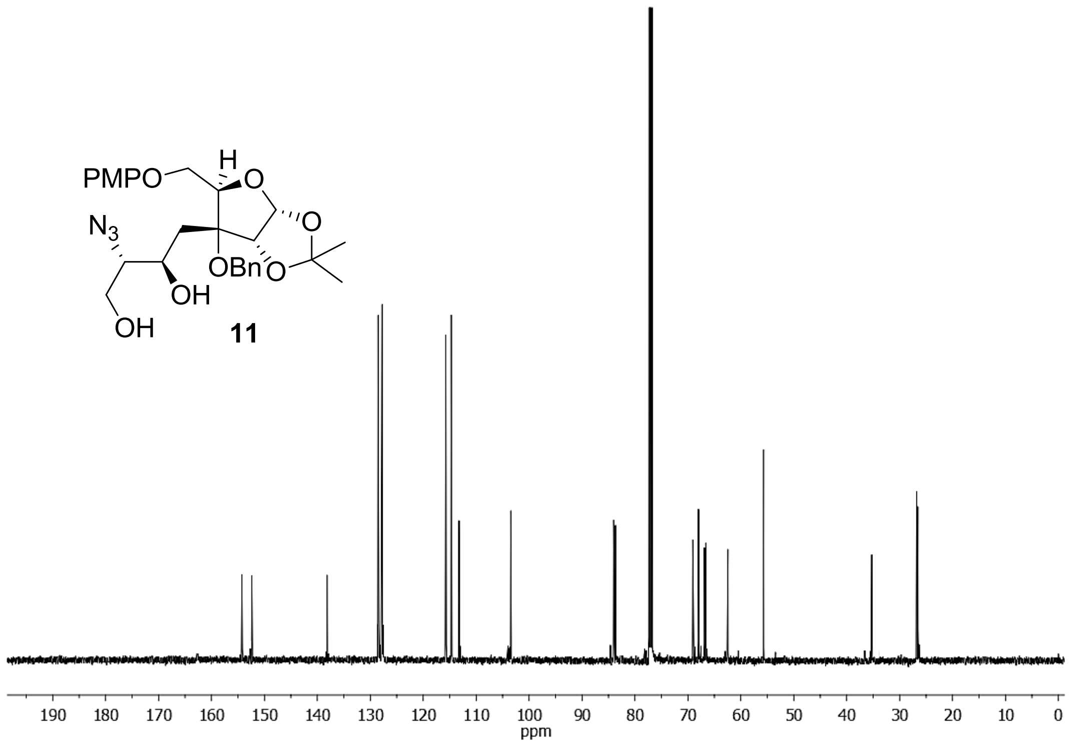


**Figure 16:** $^{13}$C NMR (125 MHz, $CDCl_3$) spectrum of compound **11**

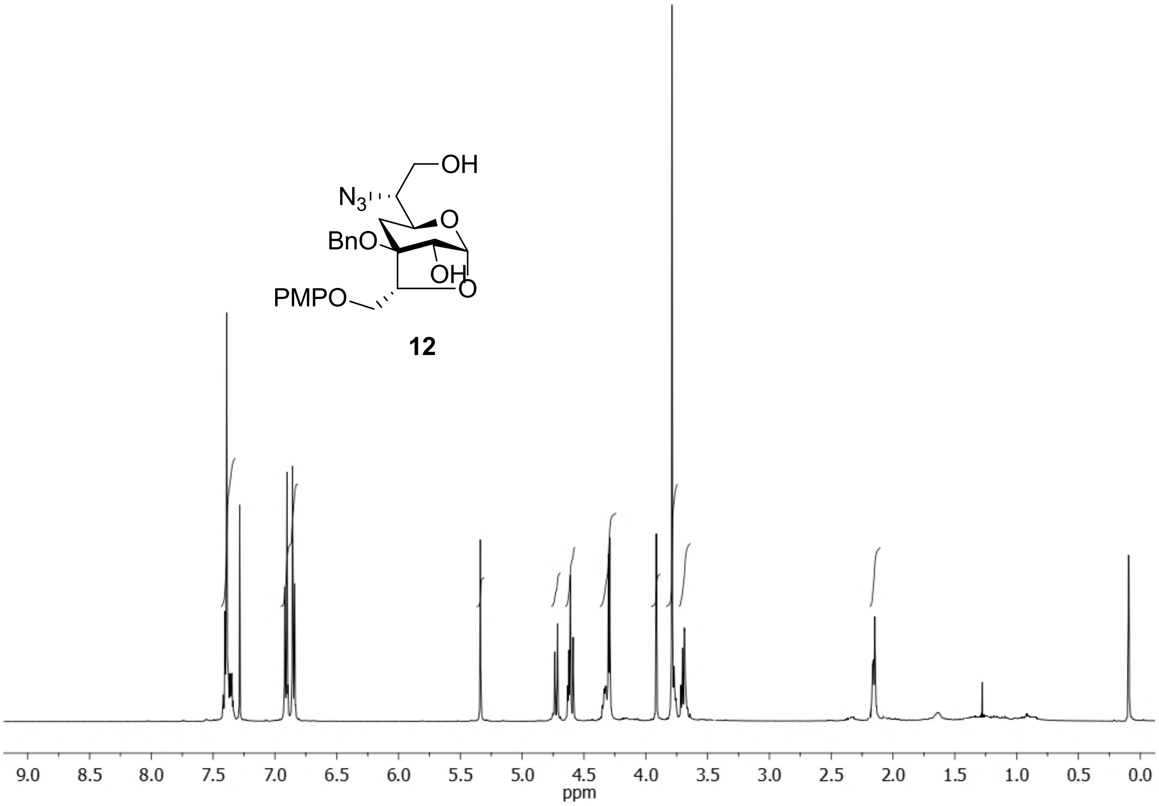


**Figure 17:** $^{1}$H NMR (500 MHz, $CDCl_3$) spectrum of compound **12**

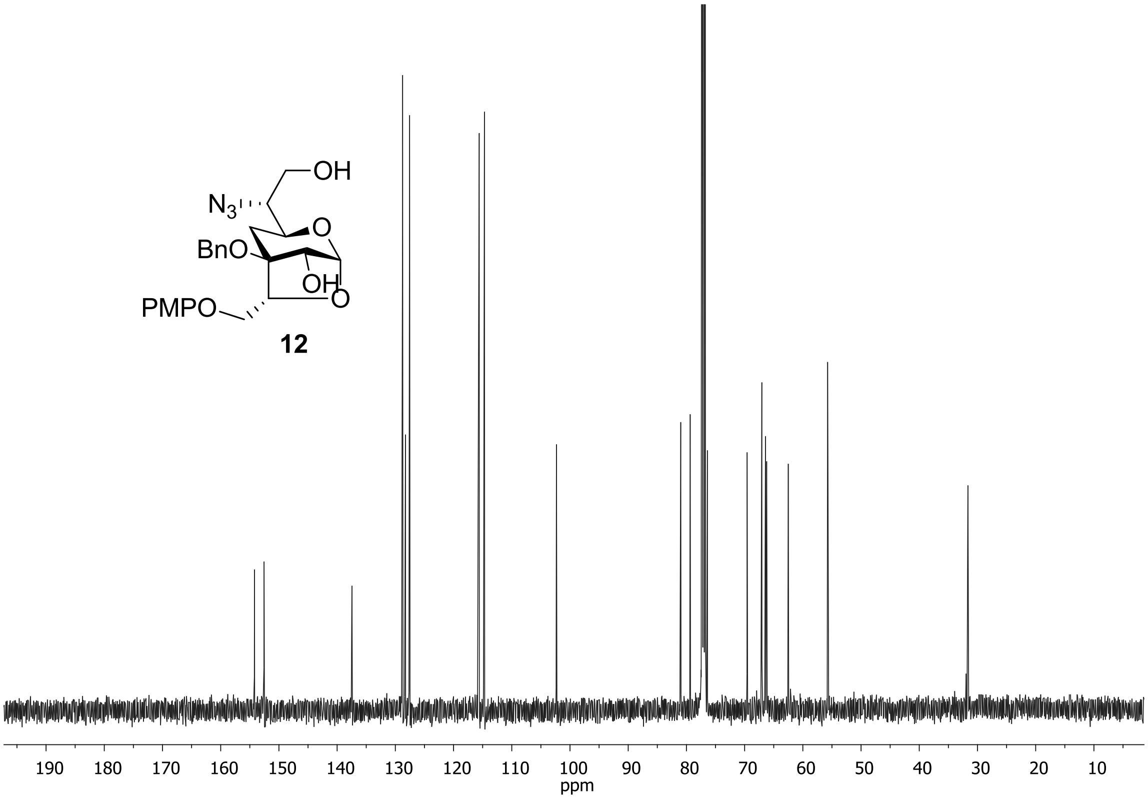


**Figure 18:** $^{13}$C NMR (125 MHz, $CDCl_3$) spectrum of compound **12**

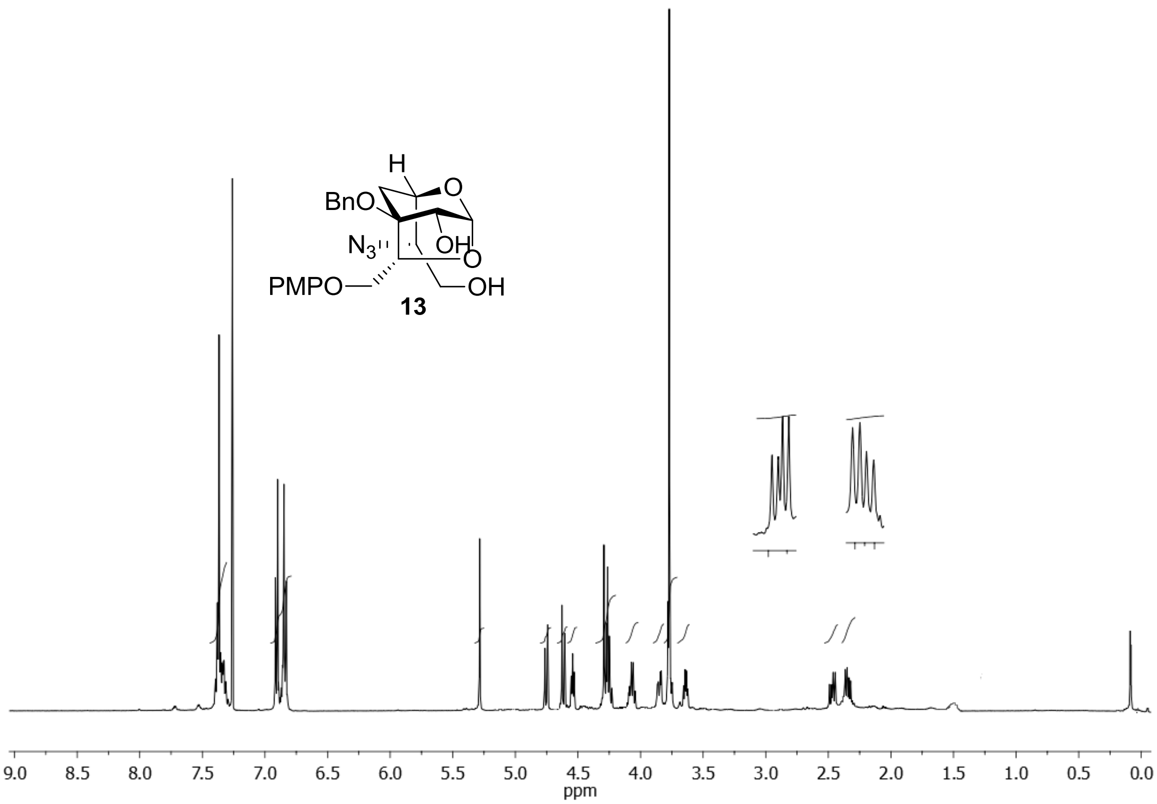


**Figure 19:** $^{1}H$ NMR (500 MHz, $CDCl_3$) spectrum of compound **13**

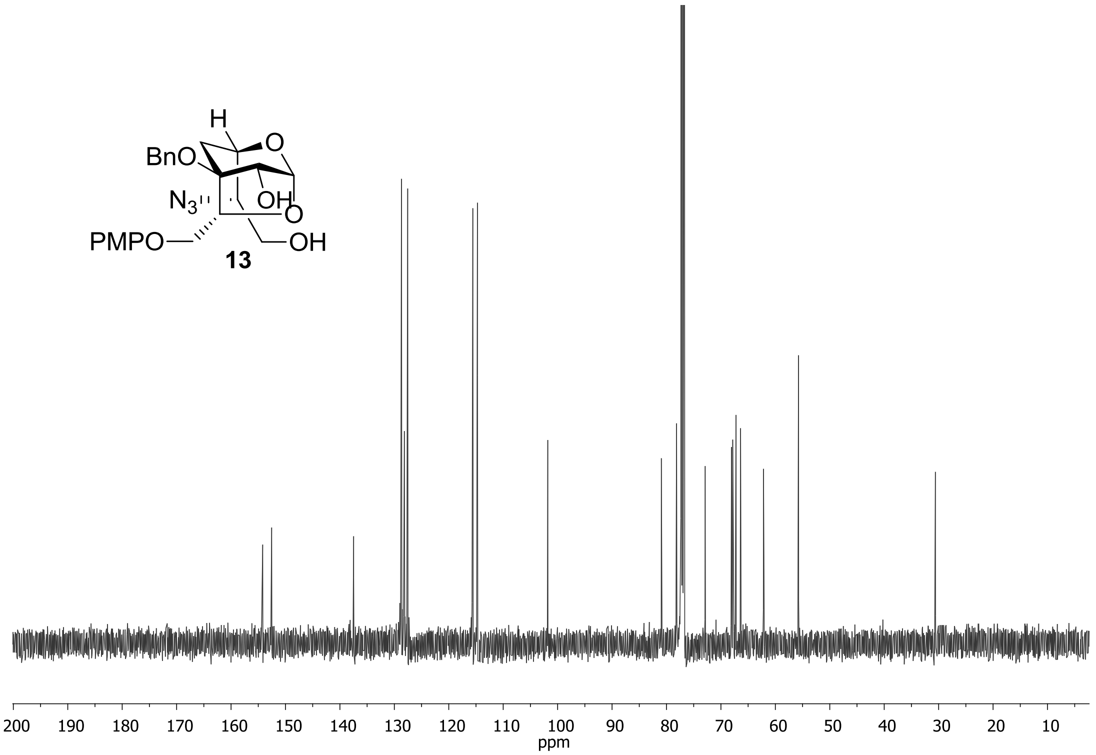


**Figure 20:** $^{13}C$ NMR (125 MHz, $CDCl_3$) spectrum of compound **13**

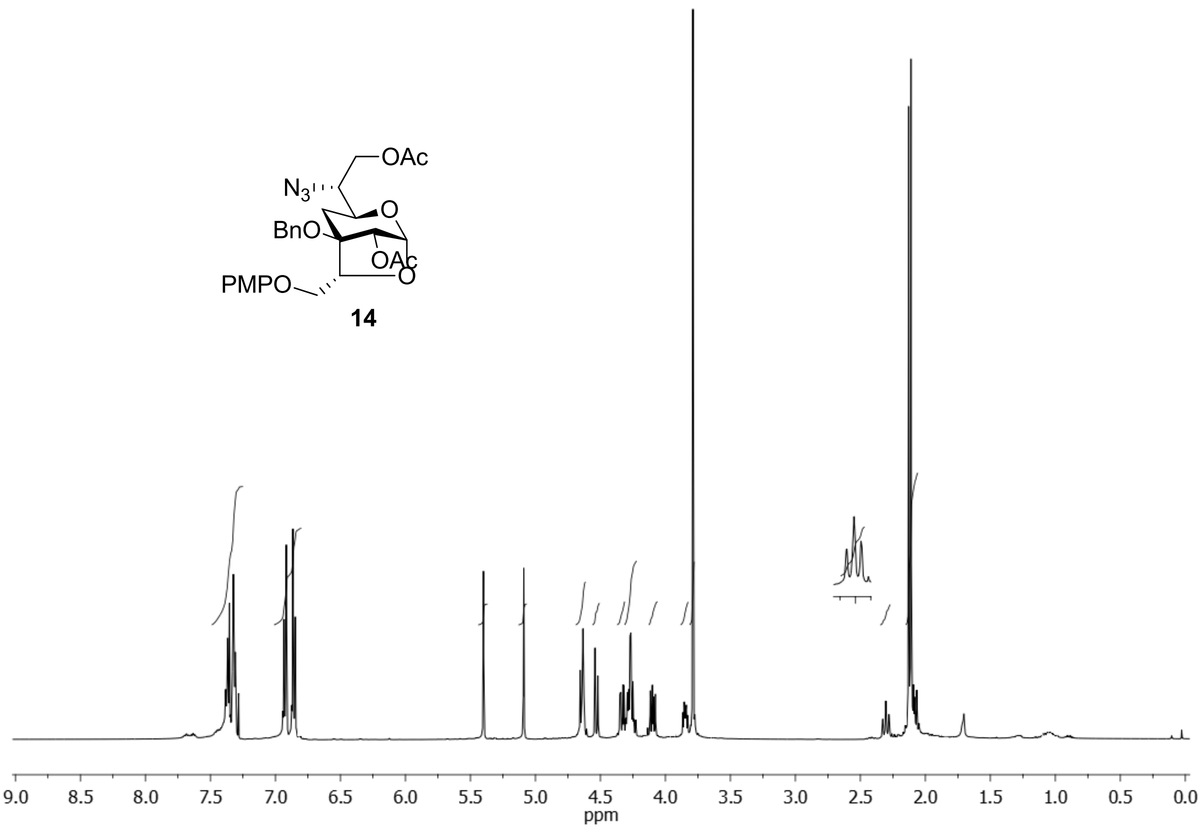


**Figure 21:** $^{1}$H NMR (500 MHz, $CDCl_3$) spectrum of compound **14**

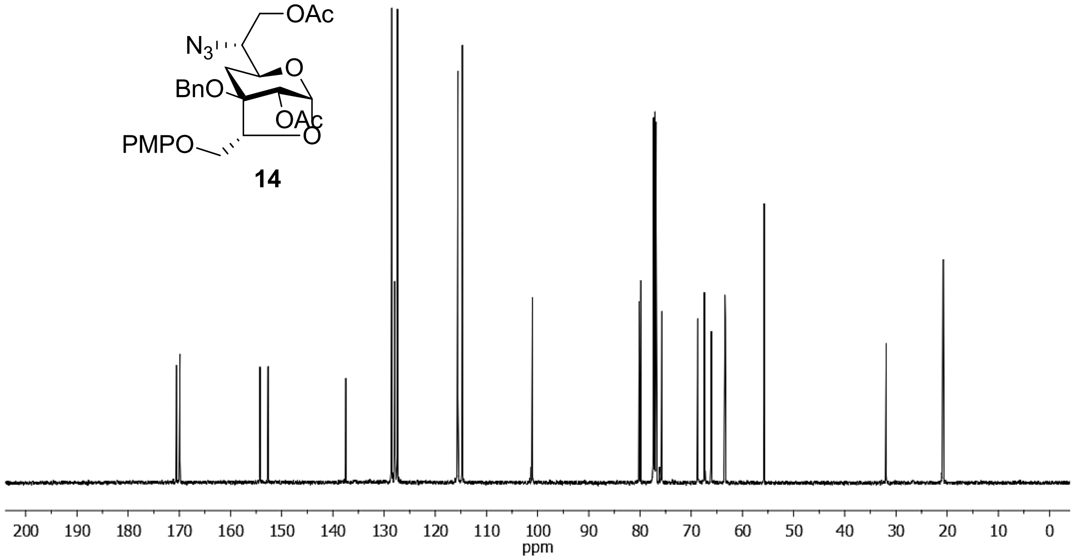


**Figure 22:** $^{13}$C NMR (125 MHz, $CDCl_3$) spectrum of compound **14**

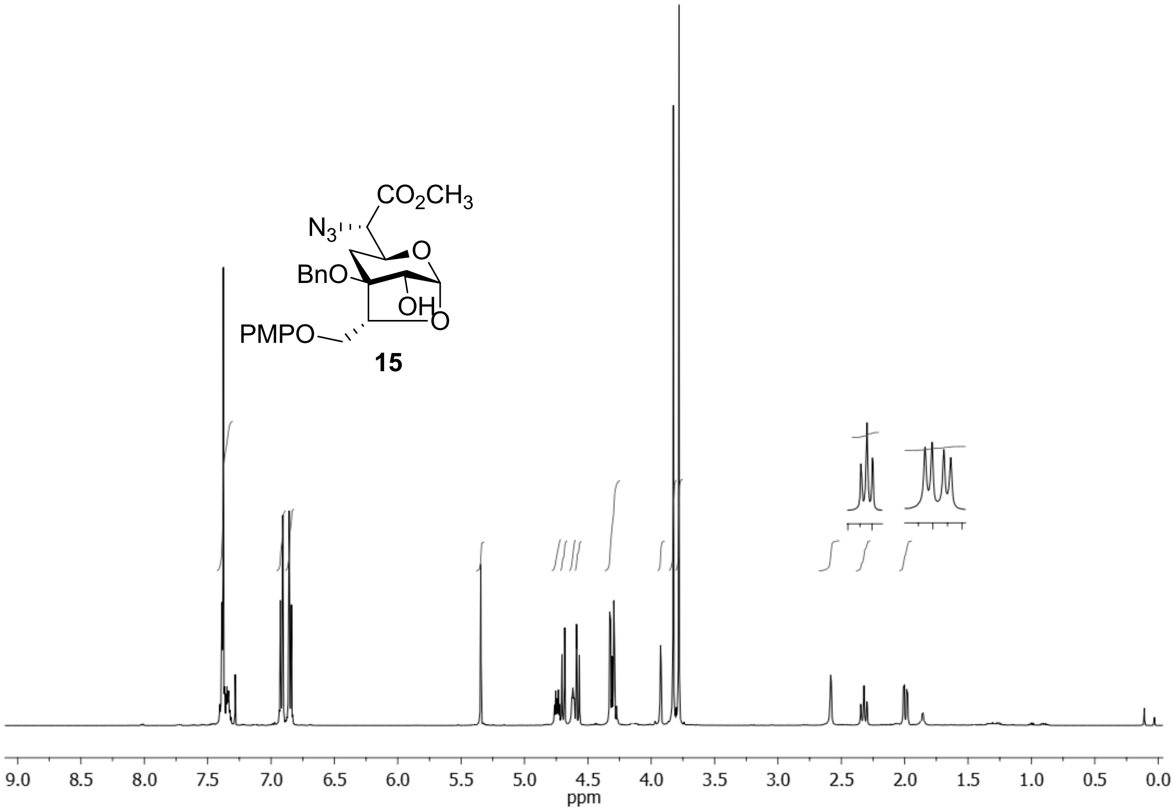


**Figure 23:** $^{1}H$ NMR (500 MHz, $CDCl_3$) spectrum of compound **15**

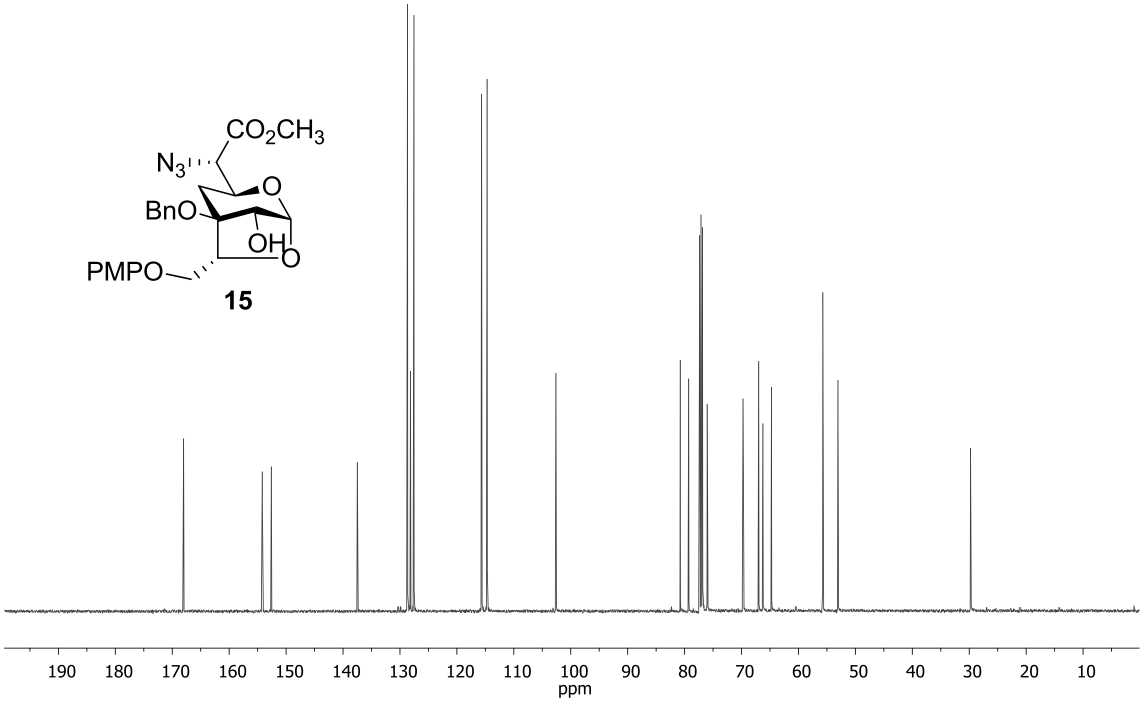


**Figure 24:** $^{13}C$ NMR (125 MHz, $CDCl_3$) spectrum of compound **15**

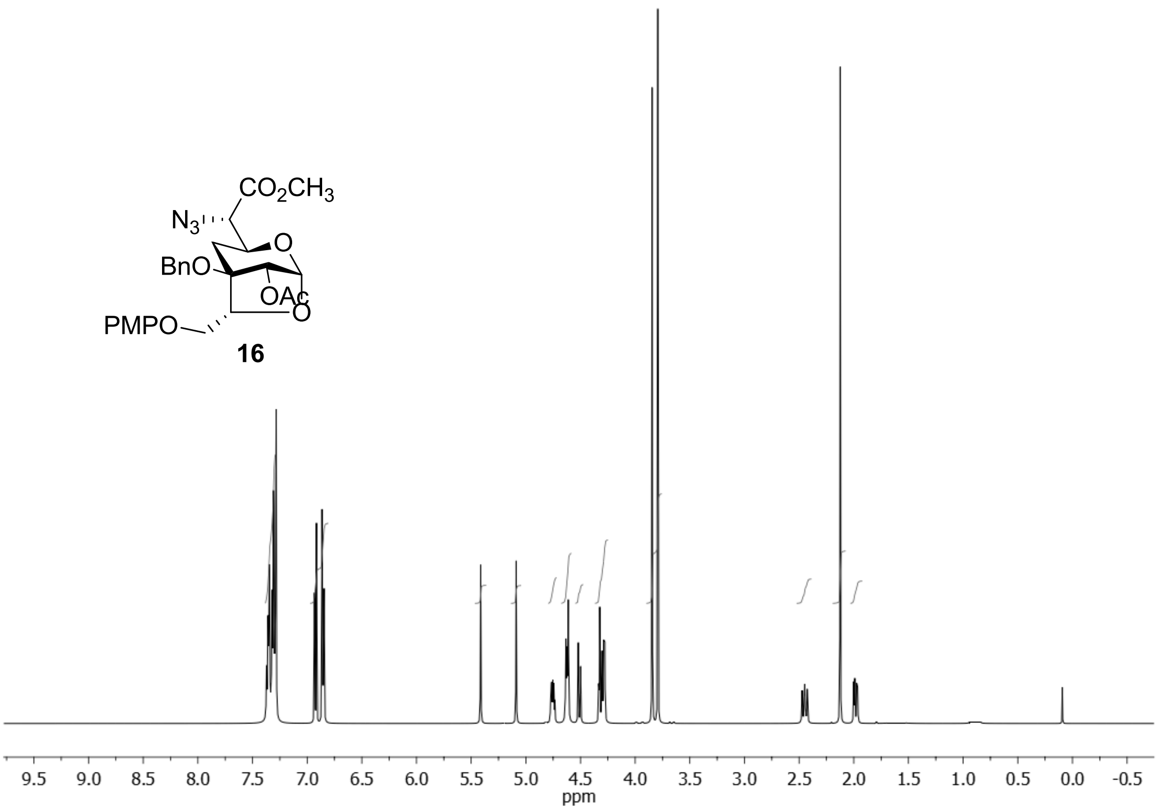


**Figure 25:** $^{1}$H NMR (500 MHz, $CDCl_3$) spectrum of compound **16**

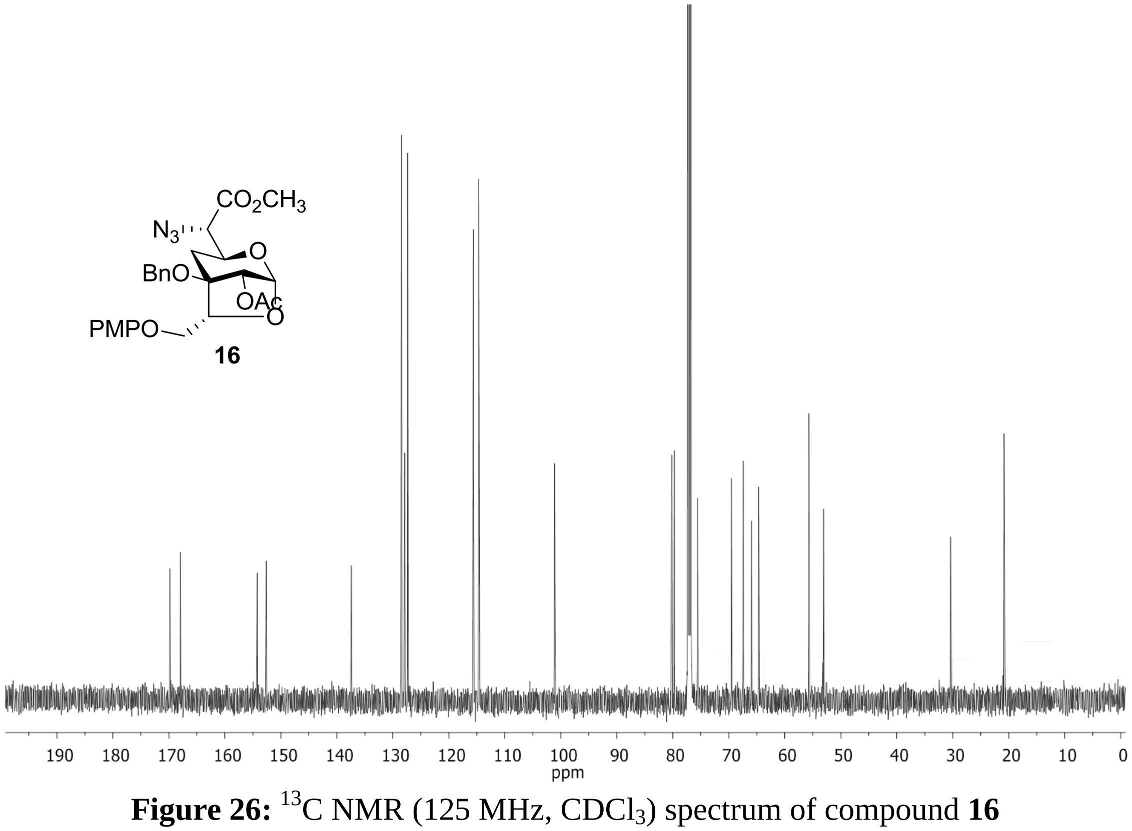


**Figure 26:** $^{13}$C NMR (125 MHz, $CDCl_3$) spectrum of compound **16**

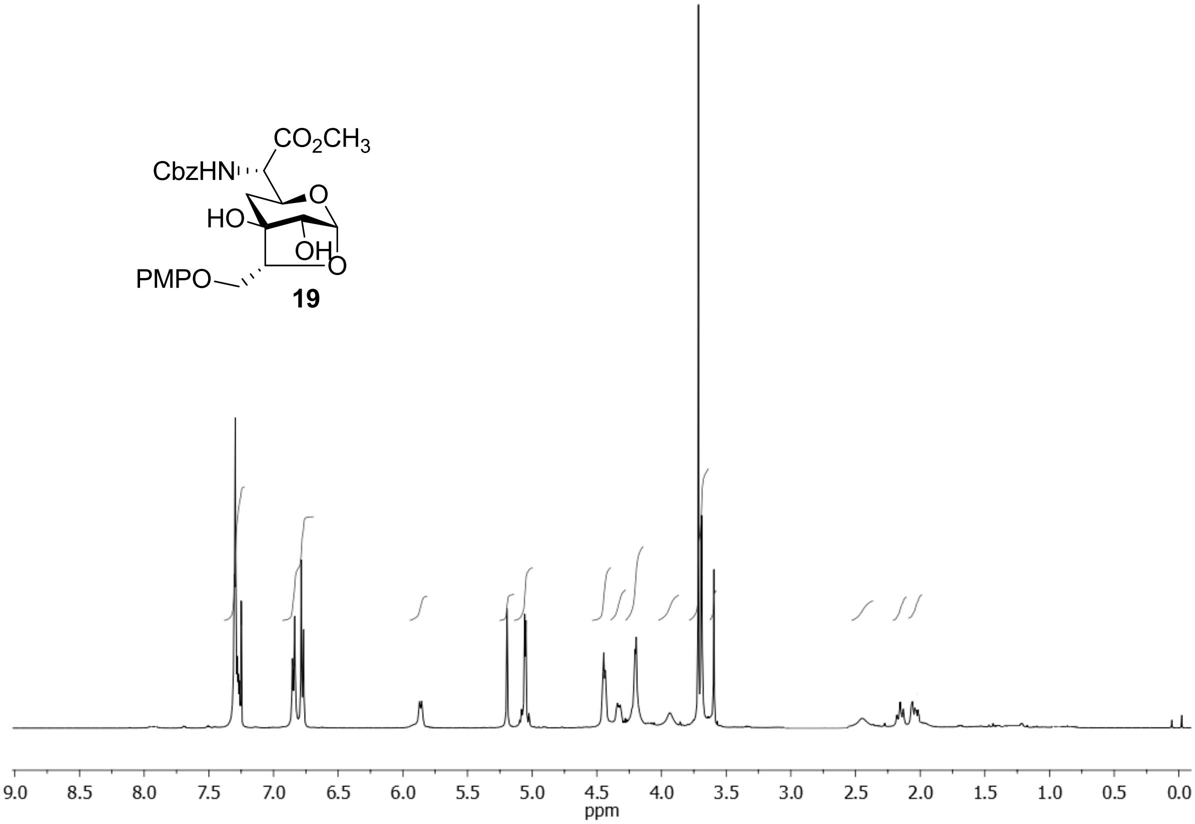


**Figure 27:** $^{1}$H NMR (500 MHz, $CDCl_3$) spectrum of compound **19**

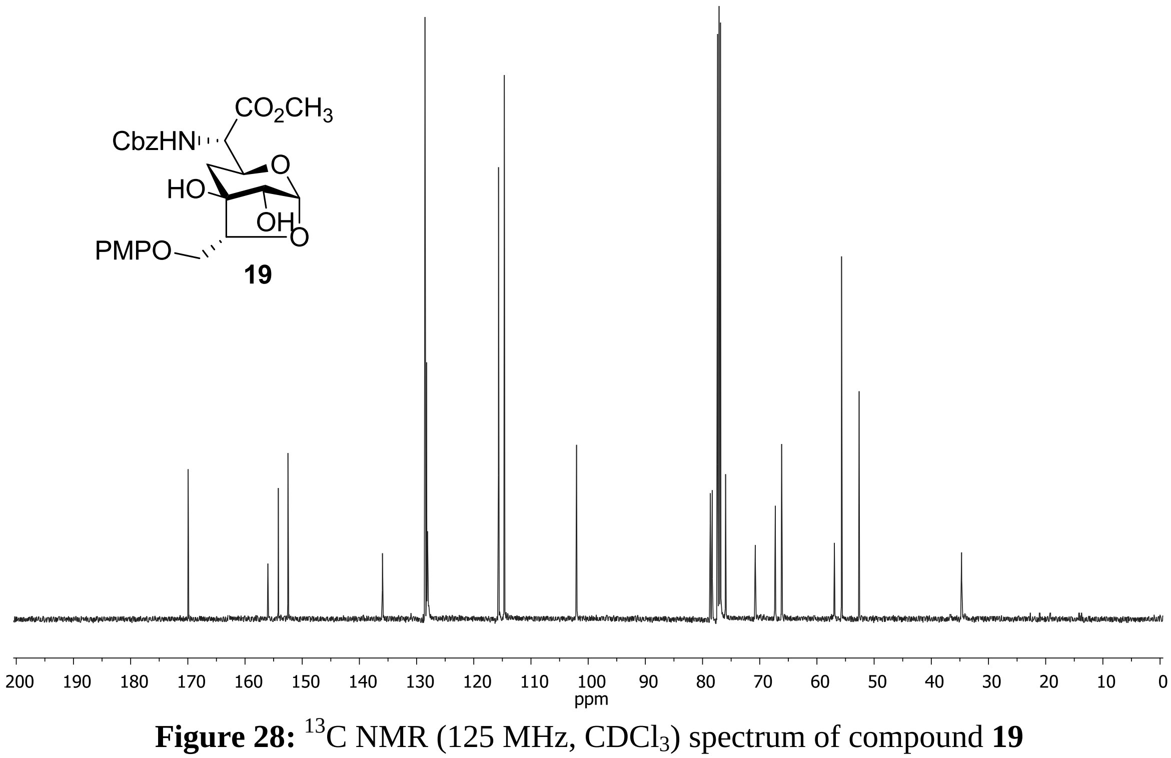


**Figure 28:** $^{13}$C NMR (125 MHz, $CDCl_3$) spectrum of compound **19**

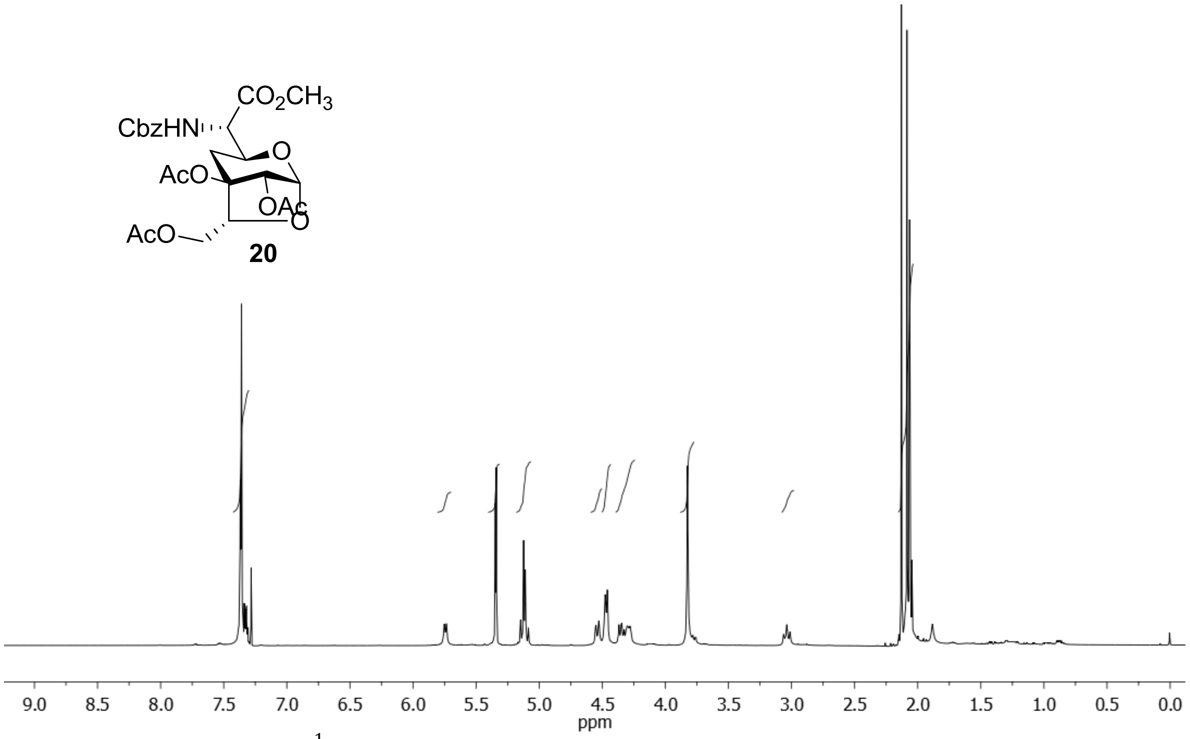


**Figure 29:** $^{1}H$ NMR (500 MHz, $CDCl_3$) spectrum of compound **20**

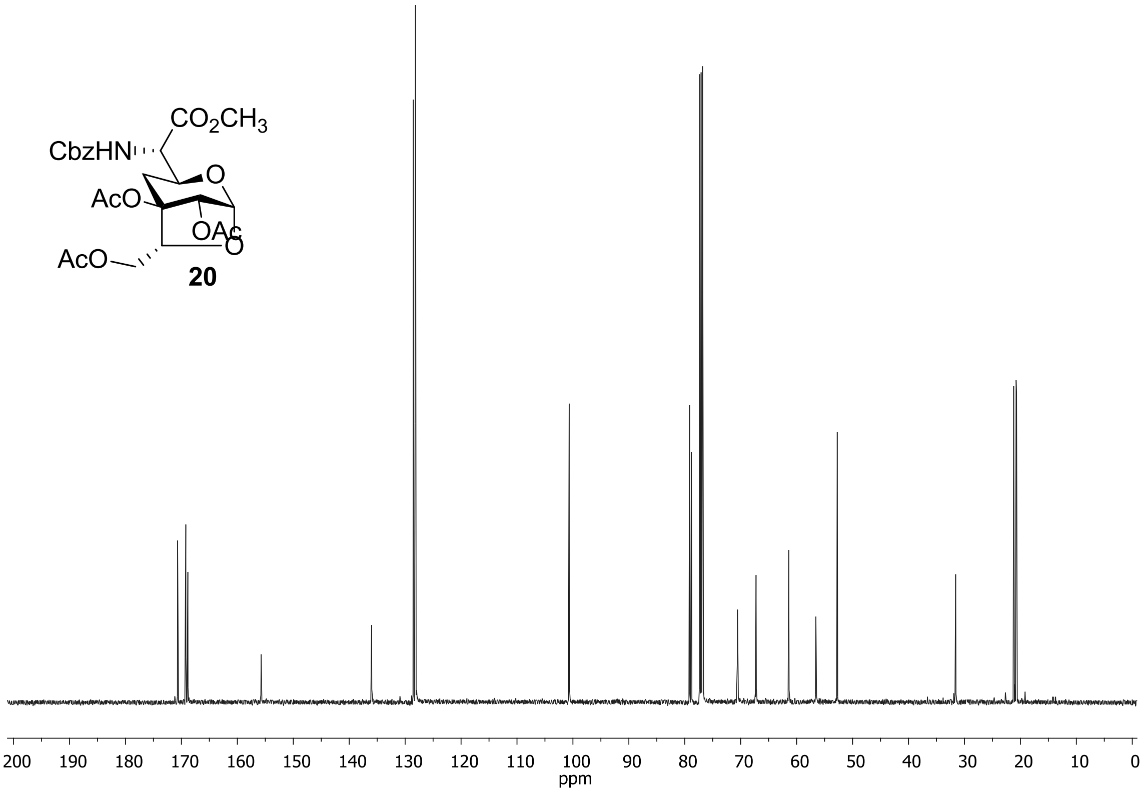


**Figure 30:** $^{13}C$ NMR (125 MHz, $CDCl_3$) spectrum of compound **20**

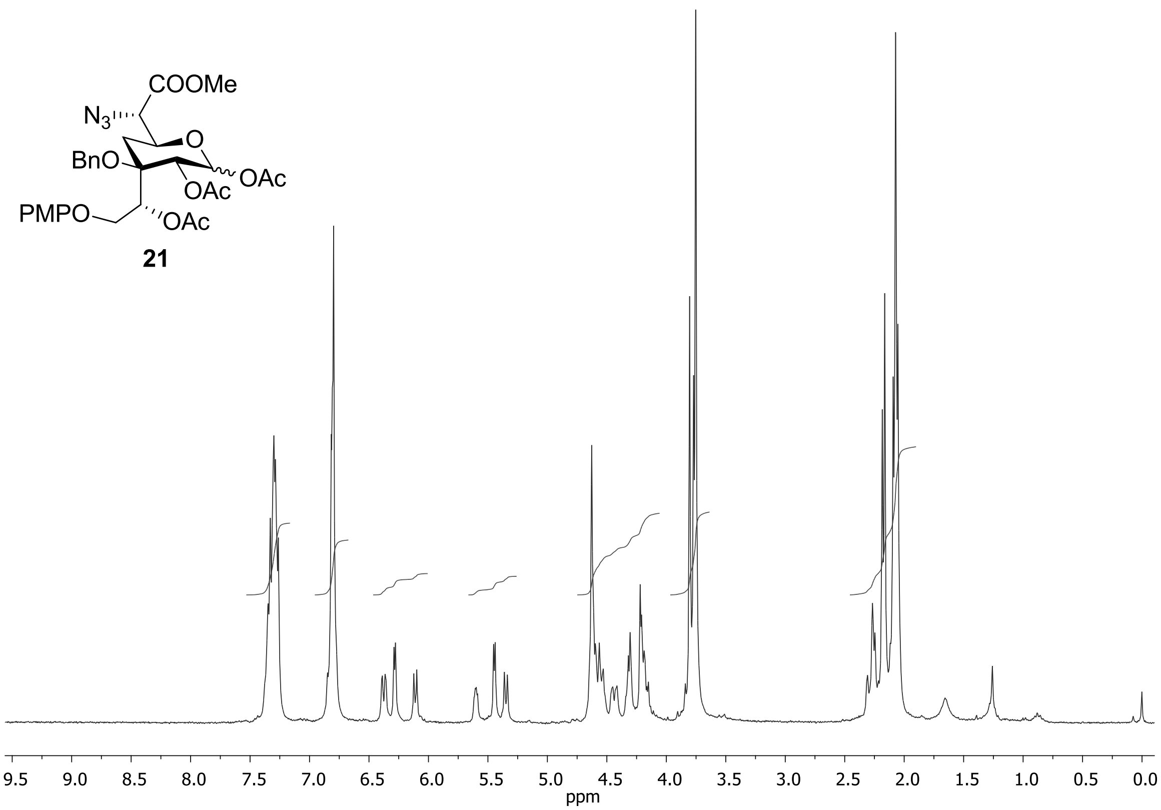


**Figure 31:** $^1$H NMR (300 MHz, $CDCl_3$) spectrum of compound **21**

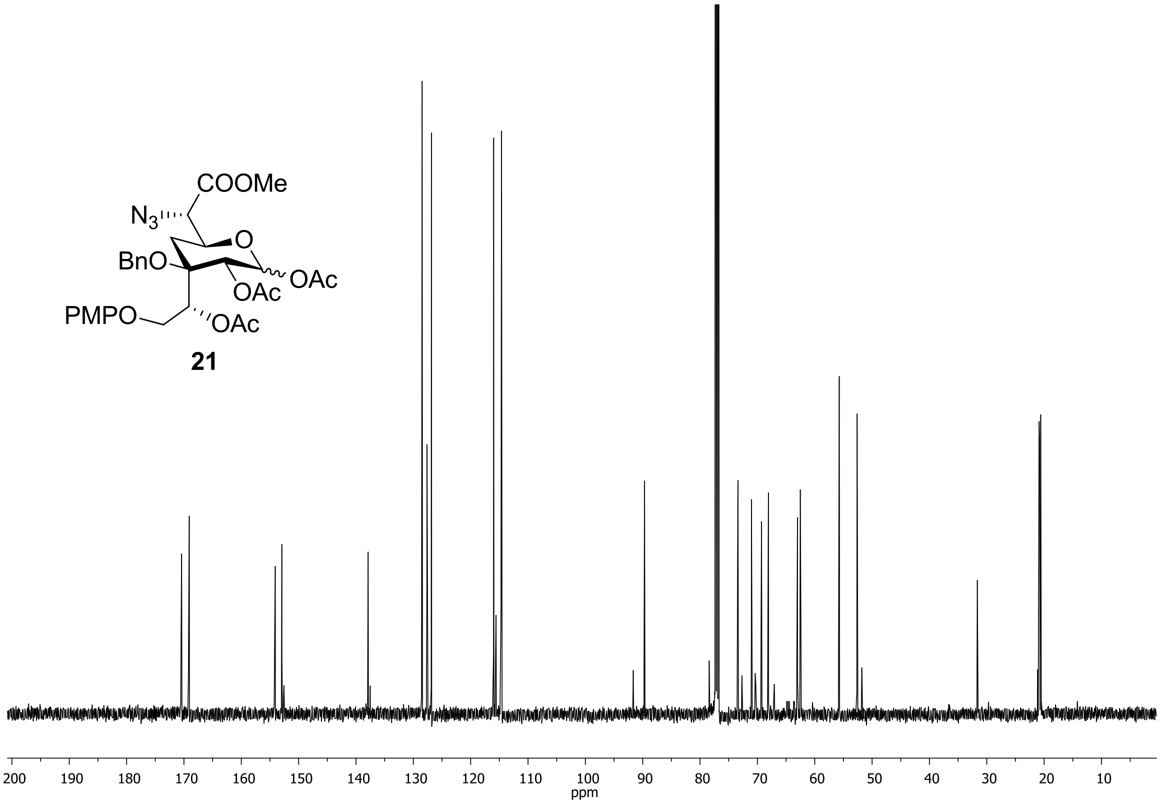


**Figure 32:** $^{13}$C NMR (125 MHz, $CDCl_3$) spectrum of compound **21**

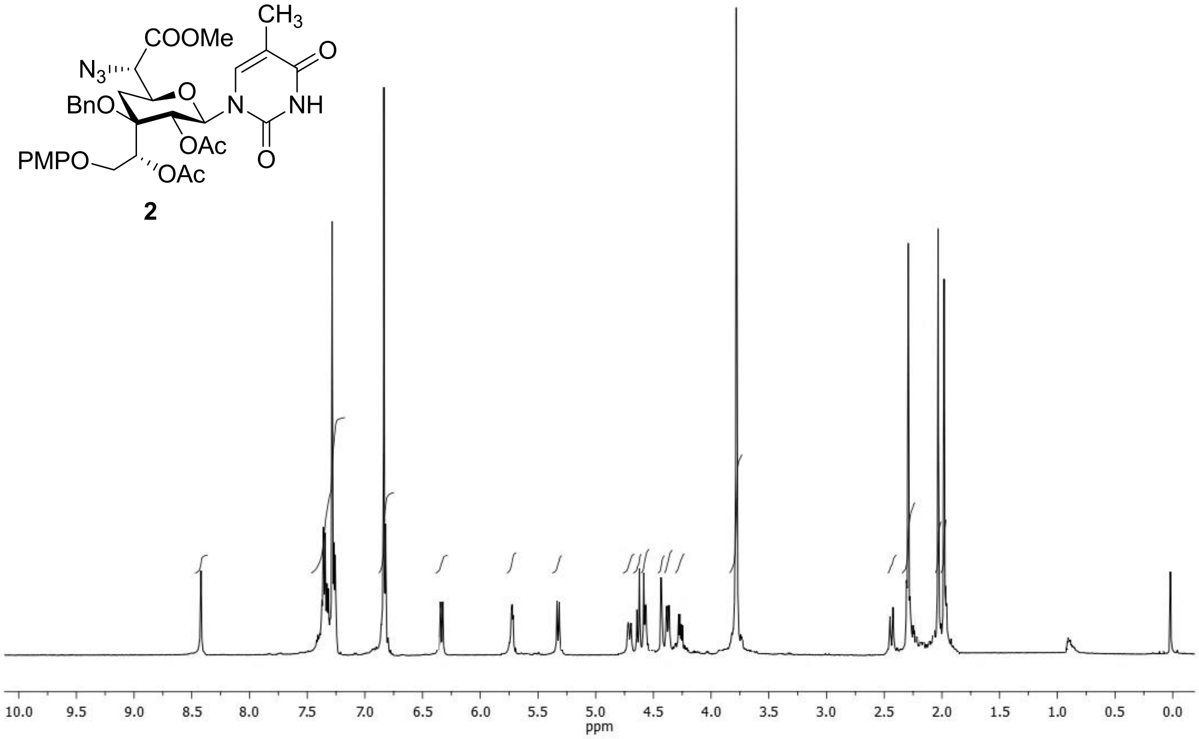


**Figure 33:** $^{1}$H NMR (500 MHz, $CDCl_3$) spectrum of compound **2**

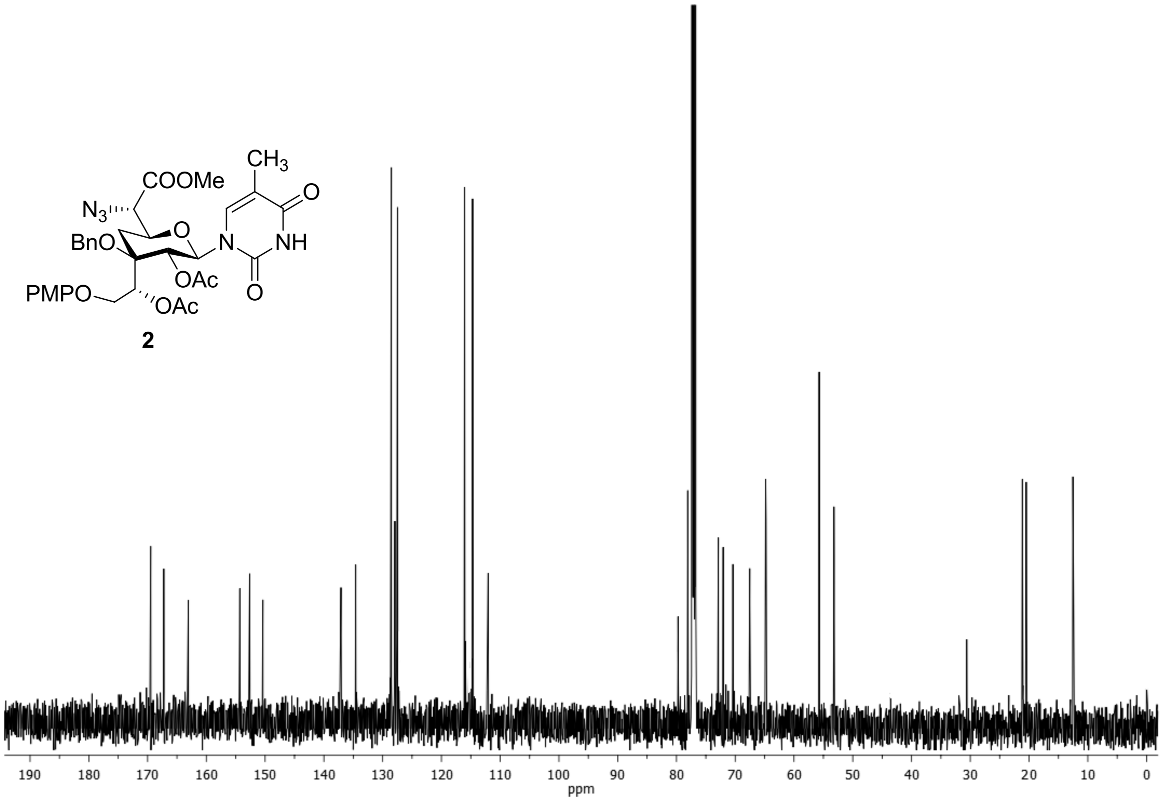


**Figure 34:** $^{13}$C NMR (125 MHz, $CDCl_3$) spectrum of compound **2**

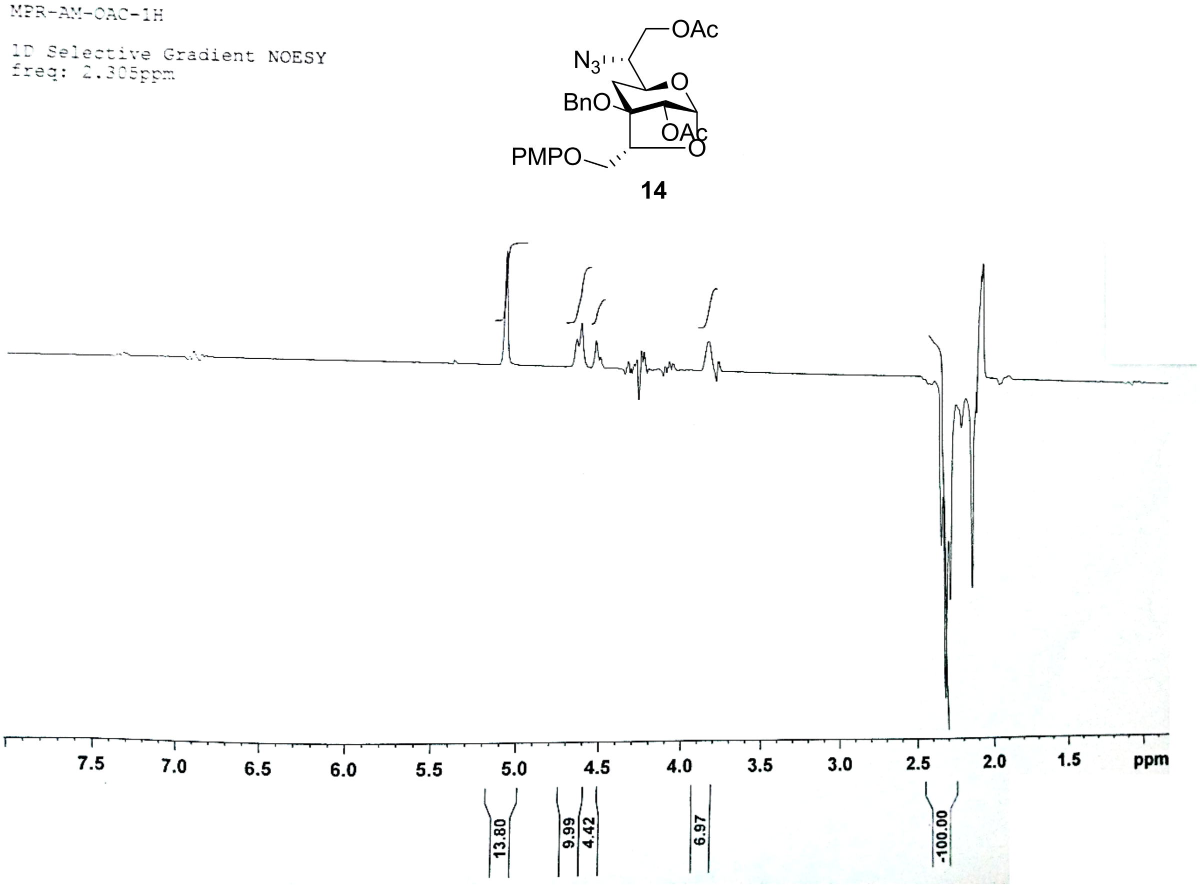


**NOE spectrum compound 14**
